# Abnormal Capillary Vasodynamics Contribute to Ictal Neurodegeneration in Epilepsy

**DOI:** 10.1038/srep43276

**Published:** 2017-02-27

**Authors:** Rocio Leal-Campanario, Luis Alarcon-Martinez, Hector Rieiro, Susana Martinez-Conde, Tugba Alarcon-Martinez, Xiuli Zhao, Jonathan LaMee, Pamela J. Osborn Popp, Michael E. Calhoun, Juan I. Arribas, Alexander A. Schlegel, Leandro L. Di Stasi, Jong M. Rho, Landon Inge, Jorge Otero-Millan, David M. Treiman, Stephen L. Macknik

**Affiliations:** 1 Barrow Neurological Institute, 350 W Thomas Rd, Phoenix, AZ 85013, USA; 2 División de Neurociencias, Universidad Pablo de Olavide, Ctra. Utrera km. 1, 41013 Sevilla, Spain; 3 Department of Neuroscience, University of Montreal, 900 Rue St-Denis, Montreal Quebec H2X 0A9, Canada; 4 Universidade de Vigo, Campus Universitario Lagoas-Marcosende, 36310 Vigo, Spain; 5 SUNY Downstate Medical Center, Depts of Ophthalmology, Neurology, and Physiology/Pharmacology, 450 Clarkson Ave, MSC 58 Brooklyn, NY 11203, USA; 6 Interdisciplinary Graduate Program in Neuroscience, Arizona State University, PO Box 874601, Tempe, AZ 85287-4601, USA; 7 University of Arizona, College of Medicine, 1501 N. Campbell Ave., PO Box 245017, Tucson, Arizona 85724, USA; 8 Graduate School of Arts and Science, New York University, New York, NY 10012, USA; 9 Sinq Systems Inc., 8070 Georgia Avenue, Silver Spring, MD 20910, USA; 10 Departamento Teoria Señal y Comunicaciones, E.T.S. Ingenieros Telecomunicacion, Universidad Valladolid, 47011 Valladolid, Spain; 11 Mind, Brain, and Behavior Research Center, University of Granada, 18071 Granada, Spain; 12 Departments of Pediatrics, Clinical Neurosciences, Physiology & Pharmacology, Alberta Children’s Hospital, Research Institute, Cumming School of Medicine, University of Calgary, Canada; 13 Norton Thoracic Institute, St. Joseph’s Hospital and Medical Center, 124 W. Thomas RD, Suite 105, Phoenix, AZ 85013, USA; 14 Department of Neurology, Johns Hopkins University, 600 N Wolfe St., Baltimore, Maryland 21287, USA

## Abstract

Seizure-driven brain damage in epilepsy accumulates over time, especially in the hippocampus, which can lead to sclerosis, cognitive decline, and death. Excitotoxicity is the prevalent model to explain ictal neurodegeneration. Current labeling technologies cannot distinguish between excitotoxicity and hypoxia, however, because they share common molecular mechanisms. This leaves open the possibility that undetected ischemic hypoxia, due to ictal blood flow restriction, could contribute to neurodegeneration previously ascribed to excitotoxicity. We tested this possibility with Confocal Laser Endomicroscopy (CLE) and novel stereological analyses in several models of epileptic mice. We found a higher number and magnitude of NG2+ mural-cell mediated capillary constrictions in the hippocampus of epileptic mice than in that of normal mice, in addition to spatial coupling between capillary constrictions and oxidative stressed neurons and neurodegeneration. These results reveal a role for hypoxia driven by capillary blood flow restriction in ictal neurodegeneration.

Progressive neuronal degeneration is a frequent consequence of prolonged and/or repetitive seizure activity^1^,^2^, and is thought to be the result of glutamate-induced excitotoxicity, which produces calcium overload and activates pro-apoptotic molecular cascades^3^. Excitotoxicity activates the same pro-apoptotic pathways as hypoxia, however, so molecular labeling of pathways underlying excitotoxicity versus hypoxia is inherently ambiguous. Local hypoxia might therefore contribute to ictal neurodegeneration. This possibility is contrary to current thinking, however, because seizure foci are macroscopically hyperemic^4^, and draining veins in the epileptic brain are hyper-oxygenated (draining veins from seizure foci turn red with oxygenated blood), both of which suggest hyperoxia rather than hypoxia within the epileptogenic focus^4^,^5^,^6^. Yet, *microscopic* hypoxia, detectable only with recent imaging techniques, might be present, even during macroscopic hyperemia and in the absence of macroscopic hypoxia.

One obstacle to determining the relative contributions of excitotoxicity and hypoxia to neurodegeneration has been that, whereas the effects of excitotoxicity can be tested *in vitro*, hypoxia due to microvascular ischemia can only be tested *in vivo*. The recent development of fiber-optic-bundle-coupled laser-scanning confocal fluorescence imaging (Confocal Laser Endomicroscopy—CLE) has made it possible to establish the contribution of microvascular dynamics to ictal neural degeneration^7^. *In vitro*^8^,^9^ and *in vivo*^10^,^11^ cortical imaging has shown pericyte-driven capillary constrictions as a function of both drug application and functional local neural activity in healthy animals^11^. No studies to date have assessed the potential contribution of hippocampal microvascular constrictions to ictal neural degeneration, however. Here we used CLE to image microscopic blood flow in the hippocampus of KCNA1-null (Kv1.1 knockout (KO)) mice^12^,^13^ (the first of the genetic rodent models to replicate human temporal lobe epilepsy (episodic ataxia type 1))^13^, and their wild-type (WT) littermates. We also turned neurotypical mice into epileptic mice by treating them with kainic-acid (KA) (a classical experimental model of epilepsy). In addition, we used two-photon laser scanning microscopy to image microscopic blood flow^14^ in the cortex of KA and healthy animals. Hippocampal and cortical vasoconstrictions occurred in both epileptic and WT animals^11^, but capillaries exhibiting microspasms were more prevalent in epileptic mice. Intravenous mural cell labeling and *in vivo* imaging showed that mural cells drove the capillary constrictions in both epileptic and healthy animals.

See Supplementary Information, Supplementary Fig. 1 for a general summary of the methods used in this study.

Because hypoxia and excitotoxicity both activate the same caspase molecular apoptotic pathway, no extant molecular label can distinguish neurodegeneration caused by hypoxia versus excitotoxicity. We therefore developed a novel stereological analysis to detect the source of the apoptosis—hypoxia vs excitotoxicity—based on the spatial distribution of oxidatively-stressed neurons with respect to the vasculature. Because hypoxia is driven by a blood flow effect (ischemia), neurodegeneration due to hypoxia should be spatially associated to vessels. Excitotoxicity is not blood-flow related and thus should not result in cell death patterns that are spatially associated with the vasculature. We found that apoptotic neurons in epileptic animals were more closely associated to the microvasculature than non-apoptotic cells, and that the apoptotic cells that were found in healthy animals were not associated with the vasculature. These results reveal a contribution of ischemic hypoxia to ictal neurodegeneration.

## Results

We recorded hippocampal capillary blood flow in awake Kv1.1 KO mice and their WT littermates. To ensure that the effects were due to epilepsy and not the specific Kv1.1 mutation in our genetic model or anesthesia, we also imaged capillary blood flow in two other epileptic mice populations: an anesthetized cohort of KO and WT mice and an awake cohort of KA treated mice (a classical model of epilepsy^15^,^16^) versus a sham group. In the awake KO and KA cohorts we also performed *in vivo* mural cell imaging of vasospasms, novel stereological methods, and cortical two-photon imaging of mural cell vasospasms to determine the effects of abnormal blood flow on ictal cell death.

### CLE of Hippocampal Capillaries *in vivo*

We used CLE to image blood flow (i.e. fluorescence in blood serum with 2MD green fluorescein dextran (Supplementary Information, Supplementary Fig. 2)) in the three different animal models. In our primary study with awake Kv1.1 KO animals, we recorded within 1154 hippocampal capillaries (703 vessels in 22 awake KO mice and 451 vessels in 19 WT littermates).

**Figure 1:**
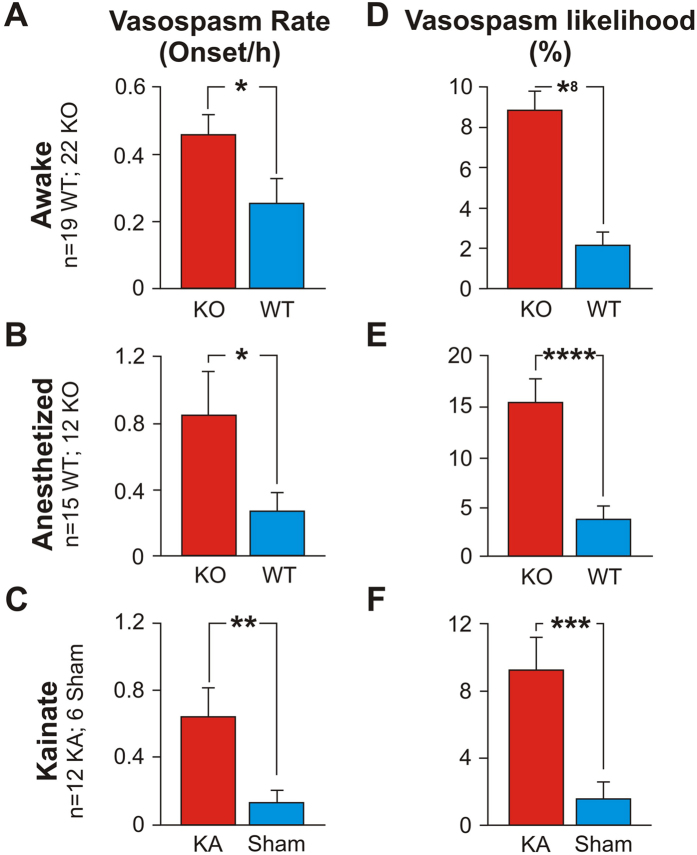
Capillary vasospasms in epileptic (KO) and WT/sham mice. Vasospasm rate ((**A–C**), number of vasospasm onsets measured per hour of recording) and vasospasm likelihood per vessel ((**D–F**), expressed as a percentage of the total recording time per vessel) were significantly higher in epileptic than in WT mice.

Vasospasms were more prevalent in the KO than in the WT animals, as shown by the following four findings:

1. The average rate of vasospasms across vessels was higher for KOs than for WTs (0.456 vasospasms/hr +/− 0.062 (s.e.m.) vs. 0.254 vasospasms/hr +/− 0.073). See Fig. 1A, expressed as the number of vasospasm onsets measured per hour of recording (t (1004) = 2.11; p = 0.035, labeled *).
2. The likelihood of a vessel exhibiting a vasospasm was higher for KOs than for WTs (8.87% +/− 0.94% vs. 2.12% +/− 0.60%). See Fig. 1D, expressed as a percentage of the total recording time per vessel (t (1100) = 6.05; p < 1 × 10^−8^, labeled *8).
3. A larger fraction of vessels exhibited vasospasms in KOs than in WTs (17.4% +/− 2.98 (s.e.m.) vs. 4.15% +/− 1.23% (s.e.m.); t (15) = 3.53; p = 1 × 10^−3^).
4. The average vasospasm magnitude was larger (4.27% +/− 0.32% vs 3.09% +/− 0.49%; t (81) = 2.00; p = 0.049, labeled *) in KO than in WT mice.

We did not find differences between cohorts for these three measures of vascular dynamics:

1. The average vasospasm duration (t (55) = 0.52; p = 0.61).
2. The average vasospasm onset speed (t (52) = 0.93; p = 0.36).
3. The average vasospasm termination speed (t (36) = −0.84; p = 0.40) (Supplementary Information, Supplementary Fig. 3).

We also imaged blood flow in 378 hippocampal capillaries in anesthetized Kv1.1 KO animals (209 vessels in 12 anesthetized Kv1.1 KO mice and 169 vessels in 15 anesthetized WT littermates). These were the initial pilot studies we conducted while developing the surgical methods to perform the deep-brain CLE recordings in awake animals. Below we show that, even in anesthetized animals, ictal vasospasms were more prevalent in the KO than in the WT mice, as indicated by the following five findings:

1. The average rate of vasospasms across vessels was higher for anesthetized KOs than for anesthetized WTs (0.846 vasospasms/hr +/− 0.262 (s.e.m.) vs. 0.273 vasospasms/hr +/− 0.107). See Fig. 1B (t (914) = 2.03; p = 0.044, labeled *).
2. The likelihood of a vessel exhibiting a vasospasm was higher for anesthetized KOs (15.42% + /− 2.33%) than for anesthetized WTs (3.75% +/− 1.35%). See Fig. 1E, (t (1064) = 4.34; p < 1 × 10–4, labeled *4).
3. We found a small difference in the fraction of vessels that exhibited vasospasms in anesthetized KOs vs anesthetized WTs (19.64% +/− 4.81 (s.e.m.) vs. 14.87% +/− 7.02% (s.e.m.); t (24) = 1.65; p = 0.58).
4. The average vasospasm duration was longer (1229.38 s +/− 183.06 vs 541 s +/− 156) in anesthetized KO than in anesthetized WT mice (t (175) = 2.86; p = 0.005, labeled 2*).
5. The average vasospasm onset speed was also greater (258.8 s +/− 44.29 vs 88.57 s +/− 40.01) in anesthetized KO than in anesthetized WT mice (t (118) = 2.85; p = 0.007, labeled 2*).

We did not find differences between cohorts for these two measures:

1. The average vasospasm magnitude (9.43% +/− 1.38% vs 10.43% +/− 3.25%; t (57) = −0.28; p = 0.78).
2. The average vasospasm termination speed (81.82 +/− 16.15 vs 108.6 +/− 38.39; t (45) = −0.64; p = 0.53) (Supplementary Information, Supplementary Fig. 3).

To determine whether these effects were due to general effects of epilepsy vs a specific function of the Kv1.1 genetic knockout, we imaged blood flow within 267 hippocampal capillaries in neurotypical mice made epileptic by injecting the KA chemical agent vs saline (147 vessels in 12 KA mice vs 120 vessels in 6 saline (sham) mice). We replicated the findings from the mutant animals, with more prevalent vasospasms in KA than sham mice, as indicated by the following four findings:

1. The average rate of vasospasms across vessels was higher for KA than for sham mice (0.642 vasospasms/hr +/− 0.18 (s.e.m.) vs. 0.133 vasospasms/hr +/− 0.068), Fig. 1C (t (885) = 2.63; p = 0.0093, labeled **).
2. The likelihood of a vessel exhibiting a vasospasm was higher for KA mice (9.87% +/− 2.20%) than for sham animals (1.74% +/− 1.05%), Fig. 1F (t (978) = 3.34; p = 0.001, labeled *3).
3. A larger fraction of vessels exhibited vasospasms in KA than in sham mice (27.02% +/− 7.79 (s.e.m.) vs. 3.35% +/− 1.83% (s.e.m.), t (12) = 2.96; p = 0.012).
4. The average vasospasm magnitude showed a small difference (6.42% +/− 1.02% vs 3.90% +/− 0.73%; t (215) = 2.01, p = 0.056) between KA vs sham mice.

We did not find differences between cohorts for these three measures:

1. The average vasospasm duration (560.3 s +/− 169.01 vs 458.05 s +/− 144.11; t (175) = 0.46; p = 0.65).
2. The average vasospasm onset speed (109.9 s +/− 31.04 vs 64.47 s +/− 14.15; t (204) = 1.33; p = 0.2).
3. The average vasospasm termination speed (51.51 +/− 27.54 vs 59.69 +/− 30.13, t (99) = −0.2; p = 0.85); (Supplementary Information, Supplementary Fig. 3).

Our results reveal only minor differences between the three animal models tested, suggesting that epilepsy leads to microvessel vasospasms in awake and anesthetized Kv1.1 mice, as well as in awake KA mice (Fig. 1B,C,E,F), and that capillary vasospasms are a general result of epileptic seizures.

Next, we analyzed the ictal vasodynamics from the awake KO cohort in greater detail.

### The Timing of Capillary Vasospasms With Respect To Seizure Onset

**Figure 2:**
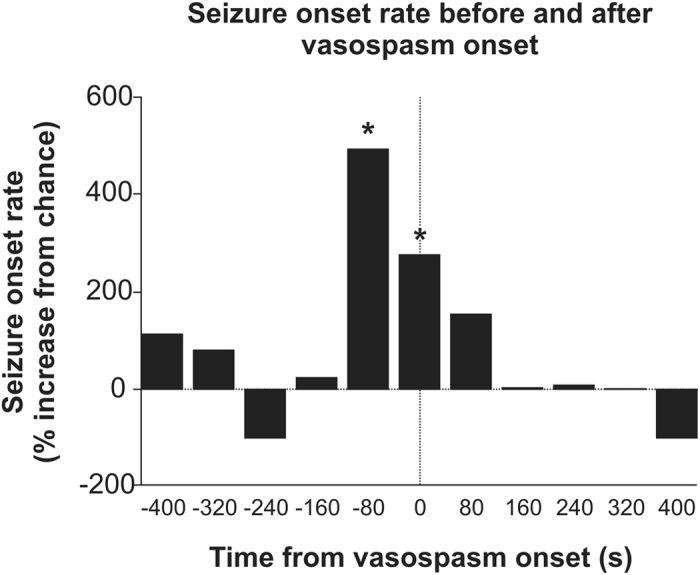
Timing of vasospasms with respect to seizure onset in awake KO mice. Vasospasm onset occurred with higher frequency (p < 0.05) within 80 secs of seizure onset (N = 703 vessels).

We examined the timing between seizure onsets and capillary vasospasms in the awake KO animals (Fig. 2). Microscopic vasospasms tended to occur within 80 seconds after seizure onset (the bins at −80 sec and 0 sec were significantly greater than chance (p = 0.0002 and p = 0.004, respectively, determined by random permutation statistics^17^). No other time points in the −400 secs to 400 secs time window reached significance). This suggests that, whereas seizures may trigger capillary vasospasms, it is less common for capillary vasospasms to lead to seizures.

### Analysis of NG2+ mural cells in Microvessel Strictures

There is a growing body of evidence indicating that NG2+ mural cells may contribute to blood flow control within capillary beds in normal function^7^,^8^,^9^,^10^,^11^,^18^. Consistent with prior observations^7^,^10^,^11^, we found capillary vasospasms in neurotypical animals (Fig. 1), suggesting that active control of normal blood flow in capillary beds may not be solely due to pre-capillary mechanisms in arteries^19^, and that capillaries also control flow directly—through both active constrictive mural cell mechanisms and passive restrictive mechanisms. Next, we examined the role of mural cells in ictal blood flow dynamics.

#### *In vivo* mural cell labeling

We developed a new selective *in vivo* mural cell labeling technique, based on the intravenous tail vein injection of 10 kD fluorescently-conjugated dextran. Previous work had shown 10 kD fluorescently-conjugated dextran to label mural cells when injected directly into the brain^20^. These prior 1 μL brain injections had produced a small number of labeled mural cells distributed randomly throughout the brain, diminishing in number as a function of distance from the injection site. We reasoned that the brain injections might have functioned by vascular uptake and transport of the dye for later deposit in distant mural cells. If that were true, we reasoned that 200 μL intravenous tail vein injections should result in the comprehensive labeling of capillary mural cells, both in WT and in epileptic animals (KO and KA mice). See Experimental Procedures for details.

#### Colocalization of capillary vasospasms to mural cells in KO mice

**Figure 3:**
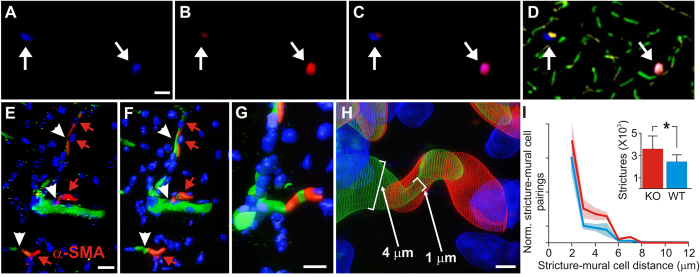
Analysis of mural cell vasoconstrictions in KO and WT mice. (**A**) Mural cells labeled immunohistochemically with anti-NG2 antibodies, and stained with a DyLight 405 (blue) secondary antibody. (**B**) Mural cells labeled *in vivo* with 10 kD AlexaFluor 647 (red) fluorescent conjugated Dextran, injected intravenously 1–6 days before sacrificing the mouse for histological processing. (**C**) Composite image of panels A & B, showing double-labeling of mural cells. (**D**) Mural cells from panels A-C, now in the context of microvasculature labeled with both 2MD *in vivo* IV injected tetra-methyl rhodamine fluorescently-conjugated Dextran (yellow), and with antibodies against endoglin protein (vasculature endothelium) tagged with Alexa Fluor 488 (green) secondary antibodies. Notice that the vessels near the mural cells have strictures (arrows). (**E**) Traditional confocal stack from KO hippocampus of immunohistochemically labeled mural cells (α-SMA, red, with red arrowheads) and vessel strictures (white arrowheads), DAPI (blue) cellular nuclei, and 2MD fluorescein dextran labeled vessels (green). (**F–H**) 3D volumetric modeling of stack in panel E (panel F), progressively magnified and rotated (panels G-H) to reveal mural cell surrounding stricture of capillary vessel. See Video S7. (**I**) Stereological quantification of hippocampal capillary strictures is significantly greater for KO than WT animals (inset), with most strictures having a paired mural cell within 2 μM distance. Scales in panels A–D = 40 μM; E,F = 10 μM; G = 8 μM; H = 1.5 μM.

To verify the selectivity of our new *in vivo* mural cell labeling technique, and determine if mural cells colocalized with vascular strictures, we employed a second immunohistochemical label, using antibodies against the mural cell biomarker NG2^21^ (Fig. 3A–D). Figure 3F–H shows a progressive zoom-in of a 3D volumetric model from a typical capillary stricture (vessel is constricted to 1 μm, down from its 4 μm unconstricted diameter), completely surrounded by a mural cell, which blocked proximal blood flow completely in this vessel. We found KO mice to exhibit 59% more hippocampal microvessel strictures than WT mice (N = 3 each cohort) (p = 0.0418, 2-tailed Wilcoxon signed rank test), see Fig. 3I (inset). Stereological distance analysis further revealed that most strictures in both KO and WT cohorts were within 2 μm of a mural cell (Fig. 3I).

#### Colocalization of capillary vasospasms to mural cells in KA mice

**Figure 4:**
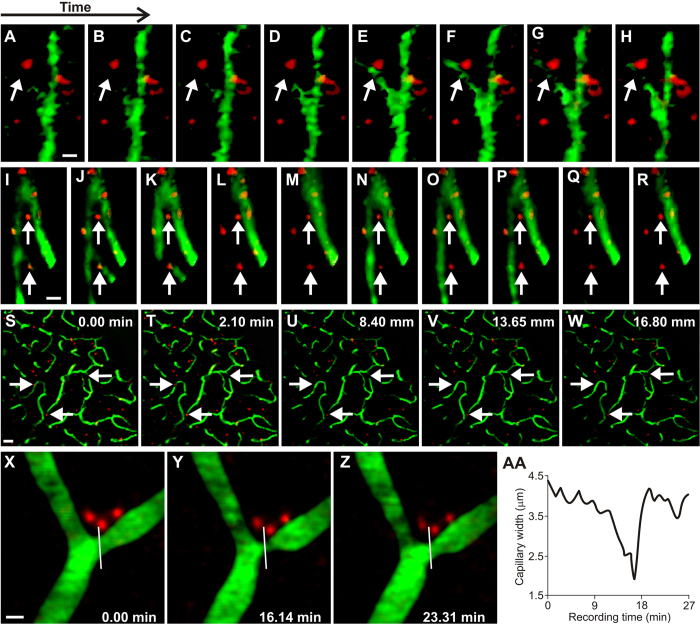
Analysis of mural cell vasoconstrictions in KA mice (**A–H**) *In vivo* fiber-optic confocal image of capillary vasospasm (vessels in green, labeled with 2MD fluorescein-conjugated dextran) colocalized to mural cells (red, labeled via intravenous vein tail injection of Alexa Fluor 647) in KA mice during seizure (arrow indicates mural cell and associated vasospasm). See Video S4. (**I–R**) Fiber-optic confocal image of capillary vasospasm colocalized to mural cells in WT mouse (arrow indicates mural cell and associated vasospasm). See Video S5 (**S–W**). *In vivo* two-photon scanning laser microscopy (TPLSM) stack of capillary vessels (green) and mural cells (red). See Video S6. (**X–Z**) *In vivo* TPLSM of a mural-cell-localized (red) capillary constriction. See Video S8. (**AA**) Quantification of vessel constriction from panels P-R measured at white line. Scales for panels A–R = 5 μM; S–W = 25 μM; X–Z = 5 μM.

KA mice afforded us the ability to image the same neurotypical animals (N = 4) both as untreated (pre-injection of kainic-acid) and treated (after KA injection) epileptic mice. We used a dual-band Cellvizio fiber-optic-coupled confocal microscope (Mauna Kea Technologies, Paris, France) to simultaneously image hippocampal vessels (injected with green 2MD fluorescein dextran) and mural cells (labeled through intravenous tail vein injection of 10 kD red AlexaFluor 647 1–6 days previous), see Fig. 4A–R. We imaged 42 capillary vessels (21 untreated, 21 after KA-injection) at labeled mural cells and observed 27 vasospasms, 13 in untreated capillaries and 14 after KA injection.

#### Two-photon imaging of mural cells in parietal cortex of KA mice

To image mural cells during vasospasms at sub-micron resolutions, we used two-photon laser scanning microscopy (TPLSM) in the parietal cortex of anesthetized mice (hippocampus is too deep for TPLSM without significant brain trauma). Because KA treatment results in generalized seizures, we reasoned that cortical capillary blood flow may present similar alterations to the ones we found in hippocampal blood flow with CLE. We imaged 635 capillary vessels (331 untreated and 304 after KA injection) in 12 mice with labeled mural cells (red) and serum (2MD green fluorescein dextran), first in the untreated animals, and then after injecting KA to induce seizures, see Fig. 4S–W. Of the 635 vessels imaged, 35 had vasospasms (5.5%; 21 in untreated animals and 14 after KA-injection), which was a lower percentage than that observed in the hippocampal recordings. The lower prevalence of cortical ictal vasospasms vs. hippocampal vasospasms may reflect the fact that the clinical presentation of ictal neurodegeneration occurs primarily in the hippocampus, rather than in the cortex^22^. Figure 4X–AA shows a mural cell constriction that was imaged with high-magnification TPLSM, revealing a slow onset and fast termination dynamics that we also saw in hippocampus (Supplementary Information, Supplementary Figs 2C and 3G–I).

### The Spatial Association Between Hippocampal Neurodegeneration And Microvessels

**Figure 5:**
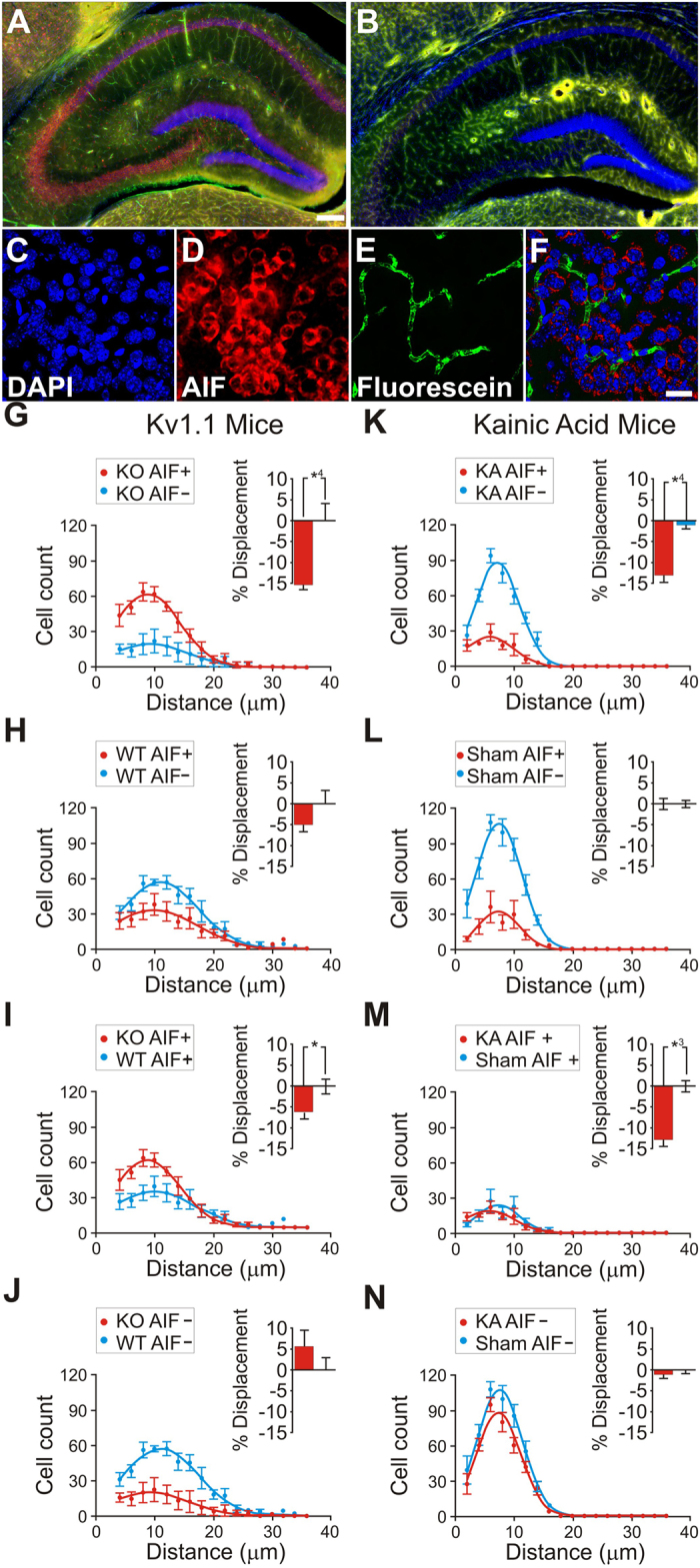
Contribution of abnormal capillary vasodynamics to neural degeneration in KO and WT mice (**A**–**F**), stereological analysis of hippocampal AIF+/− cell spatial correlation to vessels in anesthetized animals (**G**–**J**), and stereological analysis of hippocampal AIF+/− cell spatial correlation to vessels in Kainate injected (KA) animals vs sham (**K**–**N**). Immunohistochemistry of 4′-6-Diamidino-2-phenylindole (DAPI, blue), Apoptosis Inducing Factor (AIF, red), and fluorescein (green) in the hippocampus. (**A**,**B**) KO (**A**) and WT (**B**) hippocampus. (**C**) KO high-magnification DAPI stained nuclei. (**D**) AIF-labeled red fluorescence reveals AIF-positive clusters. (**E**) Green fluorescence reveals capillaries spatially associated to the AIF clusters in panel D. (**F**) Composite of (**C**–**E**). (**G**–**J**) Cell distribution as a function of distance from the nearest vessel in Kv1.1 mice, for KO AIF+/− (**G**), WT AIF+/− **(H**), KO vs WT AIF+ (**I**), KO vs WT AIF-. (**K**–**N**) Cell distribution as a function of distance from the nearest vessel in Kainic acid mice for KA AIF+/− (**K**), sham AIF+/− (**L**), KA vs sham AIF+ (**M**), KA vs sham AIF- (**N**). Scales for panels A,B = 150 μM; C–F = 10 μm. This figure is related to SI text 1, Table S2.

To quantify the contribution of abnormal capillary vasodynamics to neural degeneration we prepared sections of hippocampus from the awake KO and KA mice for histological analysis. The sections were stained with DAPI—to highlight cell nuclei in blue^23^—and with AIF primary antibodies tagged with red fluorescent Cy3 secondary antibodies—to identify cells that were oxidatively stressed and/or had engaged in apoptosis^24^,^25^ (Fig. 5A–F). Vessels had already been stained green with 2MD fluorescein dextran to prepare for CLE imaging.

We applied stereological methods to randomly sample and count neurons^26^,^27^, and developed a novel stereological technique (Supplementary Information, Supplementary Fig. 4) to measure the 3D distance between each individual hippocampal AIF+ or AIF- cell and its nearest blood vessel (Supplementary Information, Supplementary Table 1). We found that AIF+ cells were more numerous in KO mice (N = 5) than in WT mice (N = 5) (F_(3,1885)_ = 2.826, p = 0.0356) (Fig. 5G,H). AIF+ cells were, on average, 15.17% (+/−0.07% s.e.m.) closer to blood vessels than AIF- cells (χ^2^ (1, N = 1146) = 16.70, *p* < 0.0001) in KO mice, whereas there was no difference (χ^2^ (1, N = 743) = 1.976, *p* = 0.1598) in WT littermates (Fig. 5K,L). In addition, AIF- cells were not significantly further from vessels in KO mice than in WT mice (χ^2^ (1, N = 388) = 1.287, p = 0.2566), suggesting that non-degenerating cells in KO mice are normal. Finally, AIF + cells were significantly closer to vessels in KO mice than in WT animals (χ^2^ (1, N = 1541) = 5.907, p = 0.0151), supporting the idea that vascular effects are related to cellular apoptosis in KO mice.

We found similar effects in KA (N = 5) vs sham (N = 6) mice. AIF + cells were, on average, 12.683% (+/−0.804% s.e.m.) nearer to blood vessels than AIF- cells (χ2 (1, N = 2551) = 34.32, p < 0.0001) in KA mice, whereas there was no difference (χ2 (1, N = 3780) = 0.6070, p = 0.4780) in sham mice (Fig. 5K,L). Unlike in KO mice, AIF+ cells were not more numerous in KA than in sham animals: this may reflect the fact that KA animals are neurotypical for their entire lifetime up to the point of KA injection, whereas the KO animals are abnormal for their entire lifetime.

**Figure 6:**
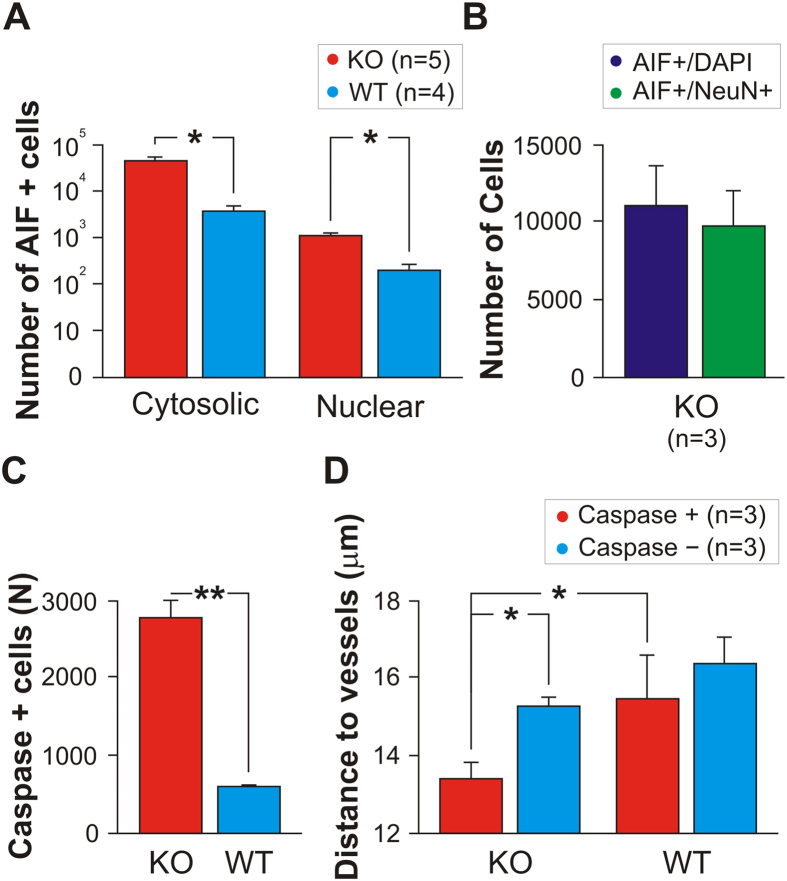
Stereological analysis of dying cells. (**A**) Number of AIF+ cells exhibiting cytosolic or nuclear labeling in KO vs WT. AIF positivity was higher in KO animals than in WT animals for both nuclear AIF staining (indicating imminent or complete cellular death) or cytosolic AIF staining (indicating oxidative stress that may lead to death). (**B**) We found that an average of 87% (+/−23%) of the AIF+ cells are Neu-N+ neurons. (**C**) Caspase positivity (indicating imminent or complete cellular death) is more prevalent in KO than in WT mice. (**D**) Caspase+ cells in KO mice lie nearer to vessels than Caspase- cells. Caspase+ cells in KO mice are nearer to vessels than Caspase+ cells in WT mice. See also SI Text 1, Tables S1 and S2.

To ensure that the oxidative stress measured with AIF affected neurons—rather than glia or other non-neuronal populations—we stained interleaved sections (from N = 3 of the originally recorded KO animals) with primary antibodies to the neuron-specific biomarker Neu-N. We found that 87% (+/−23%) of the AIF+ cells were Neu-N+ neurons (Fig. 6B). Although the AIF+/Neu-N+ count was slightly lower than the AIF+/DAPI+ cell count, suggesting that as many as 13% of the AIF+ cells may have been from non-neuronal populations, the difference was not significant.

Whereas *cytosolic* AIF positivity indicates oxidative stress in neurons^24^,^28^ (and serves as an indicator of the spatial association between oxidative stress in cells and vessels), *nuclear-translocated* AIF labeling indicates cells actively committed to apoptotic ischemic-cell death. These two labeling techniques, in combination, allowed us to determine the subset of AIF+ cells that escalate from oxidative stress to death, as follows.

First, we counted cytosolic versus nuclear AIF+ labeling (Fig. 6A), as compared to AIF- cells (in both KO and WT cohorts), and found a significant number of neurons with positivity for both cytosolic and nuclear staining (two-way ANOVA F (1, 14) = 9.70; p = 0.0076). We also found fewer dying neurons in WT than in KO mice (F (1, 14) = 7.70; p = 0.0149). Further, there was a significant interaction between AIF labeling and cohort (F (1, 14) = 7.07; p = 0.0187), in which KO mice, but not WT animals, showed a significant difference between cytosolic and nuclear AIF staining (two-tailed t (3) = 4.330; p < 0.01 (Bonferroni corrected)). These results indicate more oxidative stress leading to neuronal death in KO animals than in WT animals.

We further confirmed that AIF+ labeling indicated cellular oxidative stress and eventual death by staining interleaved sections of the same tissue with primary antibodies against active caspase-3—a pro-apoptotic molecular cascade that is AIF-independent^29^,^30^. Using stereological analyses, we found that caspase+ cells were more prevalent (two-tailed t (4) = 5.979; p = 0.0039) and nearer to vessels (two-tailed Mann-Whitney U = 52805; p = 0.0040) in KO mice than in WT mice Fig. 6C,D).

Seizures also resulted in tighter association of AIF+ cells to vessels in KA mice than in sham mice (χ^2^ (1, N = 1422) = 34.32, *p* < 0.0001) (Supplementary Information, Supplementary Table 2).

## Discussion

Our results suggest that microscopic vasospasms are more likely in the hippocampus capillary beds of epileptic mice than in those of neurotypical mice (Fig. 1). These vasospasms tend to occur within 80 secs of seizure onset (Fig. 2), and are mediated by mural cell constrictions (Figs 3 and 4). We also found that ictal neurodegeneration is spatially associated with the microvasculature, which supports the presence of ischemia-related sources of neural degeneration in epilepsy. Our analyses moreover provide a novel method to determine the relative contributions of vasoconstrictions and excitotoxicity to neural degeneration; this dissociation has been previously hindered by the fact that both of these mechanisms activate the same caspase-mediated apoptotic pathways, and hence cannot be dissociated with molecular labels (Figs 5 and 6).

The link between epilepsy and neuronal damage was first noticed almost two centuries ago^31^. Early work suggested that ictal vasospasms led to ischemia and neurodegeneration^32^,^33^, but later neurosurgical macroscopic observations found hyperemia—the opposite of ischemia—at the seizure focus^4^,^5^,^6^. Investigators concluded that seizures led to metabolic excitotoxicity, which led to cell death. Yet, cell death due to excitotoxicity activates the same apoptotic pathways as ischemic cell death, which makes it difficult to distinguish between these two potential sources of neurodegeneration. Few subsequent studies addressed the possible role of abnormal blood flow in cell death, because there were no indications of macroscopic ischemia at the seizure focus. Yet, previous research localized pockets of hypoxia within the ictal focus^34^, though the mechanism was unknown. A recent quantitative computational model found that hyperemia can exacerbate hypoxia when capillaries vasospasm, leading to heterogeneous blood flow within capillary beds^35^. Our present findings indicate that irregular capillary blood flow due to mural cell dysfunction, combined with hyperemia, can exacerbate cell death in epilepsy.

**Figure 7:**
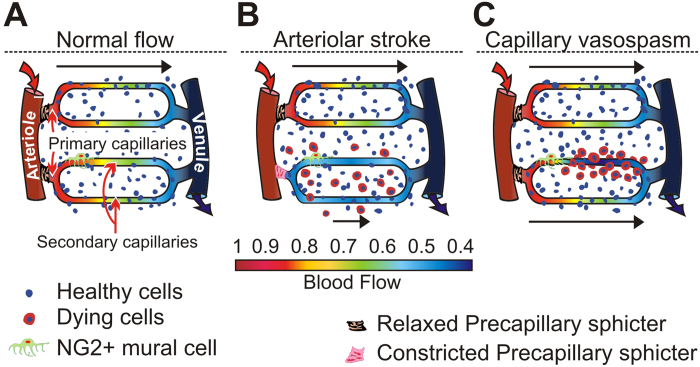
Models of blood flow control. (**A**) The prevalent model in which flow through capillaries is passive, and ultimately controlled by arteriolar structures and pre-capillary sphincters. (**B**) The prevalent model of blood flow dysfunction. An arteriolar stroke leads to uniform decrease in capillary flow within a blocked capillary bed—which leads to uniform ischemia and hypoxia—and neurodegeneration is concentrated in the watershed areas farthest from vessels. (**C**) If mural cells can non-uniformly block flow within capillary beds, in diseases such as epilepsy, neurodegeneration patterns may be altered or concentrated near blood vessels.

Tissue perfusion is thought to be lowest in the “watershed areas” between major vessels (Fig. 7A). After a stroke, watershed areas between two or more obstructed arteries are most vulnerable to ischemia because they are farthest from the blood supply. In contrast, our data indicate that after a *capillary* vasospasm, the neurons suffering the most from lack of oxygen are those *closest* to the constriction, rather than those far away from the vessel, in the watershed area (Fig. 7C). The reason—consistent with the principle that neurons farthest from the blood supply are most likely to die—may be that the inter-capillary watershed areas have the best and most redundant oxygen perfusion from unaffected capillaries, and thus the *lowest* vulnerability during seizures affecting individual nearby vessels. Thus, the same mechanism may produce opposite effects on neuronal cell death for arteriolar dysfunction (i.e. stroke) and capillary dysfunction (i.e. individual mural cell vasospasms), as a function of distance from vessels.

If this is correct, degenerating neurons affected by ischemic-hypoxic oxidative stress from capillary vasospasms should be spatially associated with the vasculature. In contrast, if excitotoxicity were the sole contributor to apoptosis, then degenerating neurons should not be spatially associated to the vasculature—neurodegeneration should be random with respect to the vasculature—because excitotoxicity is a process unrelated to the blood supply (Fig. 7B). Our data indicate a substantial vascular contribution to ictal neurodegeneration (Fig. 7C). These findings help to reconcile previous, seemingly contradictory, proposals that both excitotoxicity and vascular ischemia contribute to ictal cell death (see Supplementary Information; Supplementary Fig. 5).

Our present findings indicate that neurodegeneration in epileptic mice may result from the combination of excitotoxicity and ischemia. If this is also the case in humans, it may be possible to prevent or ameliorate the progression of neural damage in epileptic patients via the administration of blood flow regulating drugs. This could be particularly important to patients with medically or surgically intractable forms of epilepsy (i.e. patients with status epilepticus or severe forms of childhood epilepsy, including Lennox-Gastaut Syndrome, Dravet’s Syndrome, and Phelan-McDermid Syndrome), who are especially vulnerable to neural degeneration.

Mural cells contain the same KCNA1 potassium channel that is knocked out in the neurons of Kv1.1 mice^36^. Thus, one may wonder if the Kv1.1 mutation might not act on mural cells directly to produce vasospasms (which, in that case, would be unrelated to seizures). The data in Fig. 2 refute this possibility, because if the mutation drove the vasospasms, rather than the seizures, then the seizures should not precede the vasospasms; yet we found the opposite. Further, the fact that we found similar vascular dynamics in KA-treated WT animals indicates that seizures, rather than the genetic mutation per se, drive vasospasms in Kv1.1 animals.

Other alternative interpretations of our findings include: 1) excitotoxicity may injure not only neurons, but also non-neuronal populations such as blood vessels, mural cells, or astrocytes (which, if damaged, could produce microvascular dysfunction and increased rates of capillary vasospasms) and 2) our stereological measures of degeneration may have mistakenly counted non-neurons as neurons. We addressed these issues by performing Neu-N controls: these indicate that our stereological measurements did not mistake non-neurons for neurons in large numbers (most—if not all—of the degenerating cells were labelled by Neu-N and therefore were neurons).

Some or all of the mural cells identified in our study may be either pericytes or smooth muscle cells (SMCs). A recent study suggests that traditional molecular biomarkers of pericytes (such as anti-NG2 anti-bodies) may also label smooth muscle cells^37^. We are agnostic on the issue, as, either way, it is not relevant to our finding that epilepsy results in inhomogeneous flow within microvessels—driven by capillary mural cells—that leads to vascularly-associated neurodegeneration.

## Materials and Methods

In conducting research using animals, the investigators adhered to the laws of the United States and regulations of the Department of Agriculture. All procedures were approved by the Barrow Neurological Institute Institutional Animal Care and Use Committee and performed in accordance with the NIH Guide for the Care and Use of Laboratory Animals.

We used a total of 124 mice for these experiments. We genotyped all mice descended from mutant breeders (Transnetyx Inc. USA and/or in-house). In the fiber-optic confocal blood flow imaging experiments we used 41 awake KCNA-null Kv1.1 knockout (KO = 22) and wild-type (WT = 19) littermates, 27 anesthetized mutant mice (12 KO, 15 WT littermates), and 18 neurotypical C57BJ kainic acid (KA) treated (12) vs sham (6) animals (Fig. 1). We used 4 KA-treated mice (imaged both before and after KA injection) for the dual-band Cellvizio hippocampal mural cell imaging (as in Fig. 4; Mauna Kea Technologies, Paris, France), and 12 KA mice for the TPLSM cortical mural cell recordings. For some of the stereology studies we used brains taken from a subset of the animals imaged above: five of the KO, five of the WT, six of the sham and five of the KA.

Mutant breeders were obtained from Jackson Laboratories (Bar Harbor, Maine), and were bred in-house and genotyped (either in-house or by Transnetyx Inc., USA) for use in these studies. The Kv1.1 KO mouse is a clinically relevant model of partial-onset epilepsy for the following reasons: 1) seizures manifest in the early postnatal period, corresponding to early childhood in humans, and are likely of limbic origin; 2) progressive histological changes in the hippocampus of these mice are similar to those observed both in human epileptic tissue and in many animal models of temporal lobe epilepsy; and 3) the KCNA1 gene—which encodes the delayed rectifier potassium channel alpha subunit Kv1.1—is one of only a few epilepsy genes in a rodent model that has a homologue in a human epileptic condition^13^. All mice were maintained on a 12 h light/dark cycle. Food and water were available *ad libitum*. All efforts were made to minimize the discomfort and number of animals used.

Before imaging, we injected fluorescein-coupled dextran (see Supplementary methods) and positioned the fiber-optic objective (Cellvizio, Mauna Kea Technologies, Paris, France) into the hippocampus of awake spontaneously seizing mice to visualize blood flow dynamics during EEG-determined normal, ictal, or inter-ictal periods of neural activity (Supplementary Information, Supplementary Figs 1, 2, 6 and 7).

To determine whether ischemia contributes to ictal neural degeneration, we labeled fixed tissue with immunofluorescent labels of cellular nuclei (DAPI), the neuronal marker Neu-N, antibodies against alpha smooth muscle actin (α-SMA) to visualize mural cells, and indicators of oxidative stress and apoptosis (such as antibodies against Apoptosis Inducing Factor and Caspase-3). We conducted standard stereological analyses and also developed a novel 3D stereological nearest-neighbor probe to determine if this abnormal blood flow resulted in neural damage (Figs 3, 5, 6 and 7). To visualize mural cells that constricted vessels, we developed a novel *in vivo* mural cell fluorescence labeling system and used a combination of dual-channel fiber-optic-coupled imaging system in the hippocampus and high spatial- and temporal-resolution TPLSM (Prairie Technologies, Madison, WI) in the cortex to directly visualize microvessel constrictions during seizures and normal function. We created a 3D volumetric digital model of mural cell constrictions from the labeled tissue.

### General surgical methods

Before implanting a craniotomy chamber and head-holder, we anesthetized the animals with Ketamine-Xylazine (100 mg/kg–10 mg/kg i.p.), continuously monitoring and controlling body temperature with a heating blanket and a rectal thermometer (TC-1000, CWE Inc., USA). Skull perforations were performed to insert epidural EEG recording electrodes (Supplementary Fig. 6; red and grey dots) as well as the craniotomy over the right hippocampus for the fiber-optic bundle used in the confocal microscopy (Supplementary Fig. 6A, red rectangle). A robotic stereotaxic drive (StereoDrive, Neurostar GmBH, Germany) implanted a 300 μm beveled fiber-optic bundle (5000–7000 3 μm-wide fibers) into the hippocampus (Supplementary Figs 1 and 7), and green fluorescein lysine-fixable dextran (2MD, Invitrogen, USA) was tail-vein injected (1 ml/kg of Fluorescein 5% w/w) to visualize blood flow.

### Fiber-Coupled CLE Image Analysis

We assigned a region-of-interest (ROI) to each vessel in each movie and analyzed the changes in fluorescence as a function of time. We monitored EEG to detect seizures continuously (Supplementary Information, Supplementary Figs 1 and 6). We used two Cellvizio fiberscopes (a single-band Leica Microsystems, GmbH, Germany FCM-1000 fiber-optic confocal microscope manufactured by Mauna Kea Technologies emitting at 488 nm; and a dual-band Cellvizio emitting at both 488 nm and 660 nm) with 300-micron penetrating fiber-optic probes precisely positioned surgically in the hippocampus of each mouse with a Stereodrive 3-axis robotic stereotaxic (Neurostar GmbH, Germany). On-board image analysis software was used to create ROIs of each recorded vessel. From this we derived an independent measure of each vessel’s fluorescence-over-time (ΔF/F). From these fluorescence measures we calculated the rate of vasospasms, the percentage of time that vessels vasospasmed, individual vasospasm duration, vasospasm magnitude, and onset and termination speeds (Fig. 1). The experimenter who collected the data was not blind because only the KO mice have spontaneous seizures, thus she could not be blinded from identifying KO or WT by animal’s behavior or EEG. We therefore controlled for experimenter bias by keeping the image analyst blind to the cohort. In addition, this blind analyst measured vasospasm internal dynamics automatically and objectively via custom MATLAB (Mathworks, Natick, MA) software. We averaged the metrics across animals in each cohort and tested the significance of the difference between cohorts with standard two-tailed unpaired Student’s t-tests.

We assessed the seizure rate, as a function of vasospasm onsets, separately for each KO mouse. We determined the pooled chance probability of seizure onset—the baseline seizure rate—by shuffling the seizure onset times with 10,000 random permutations, and assigning the resultant correlation to vasospasms as baseline (0% level) in the analysis. We then correlated the actual seizure times to vasospasms to create a histogram of normalized seizure onset rates as a function of vasospasm onset time.

### Mural Cell Labeling Method

Our mural cell labeling method builds on a previous study that showed that mural cells could be selectively labeled by direct 1 μL brain injections of fluorescent dextran^8^. To determine the effectiveness of our novel mural cell label, and to stereologically measure the distance between mural cells and vascular strictures in fixed tissue, we created tissue for traditional confocal and stereological analysis in KO (N = 3) and WT animals (N = 3) that were IV injected with our mural cell label (2MD fluorescein dextran to label vessels in green). We sacrificed the animals 1–6 days post-injection (to allow for mural cell labeling), processed the hippocampal tissue histologically (see Immunohistochemical procedures section below), labeling cellular nuclei in blue with the DNA-binding dye, 4′,6-diamidino-2-phenylindole dihydrochloride (DAPI)^23^, and labeled mural cells immunohistochemically in red with anti-α-smooth muscle-actin primary antibodies (α-SMA) with Cy3 secondary antibodies^9^, see Fig. 3E. We could have potentially used a transgenic mouse with a fluorescent protein tagged to NG2, but by developing a new injectable label we were free to then use it in wild-type animals, or in mutants and/or chemically treated animals with epilepsy. We injected 200 μL of fluorescent dextran (0.5 mg of dye in 0.2 ml of ACSF) intravenously (tail vein), which labels mural cells within 16–24 hours, as we verified with double-labeling by anti-NG2 Chondroitin Sulfate Proteoglycan antibodies in subsequent histological studies (Fig. 3A–D). Labeling lasts at least 6 days (the longest post-injection duration we tried). The label functions well in adults but fails in juveniles P21 or younger for unknown reason. Some mural cells endocytose the dye into vacuoles (as in Fig. 4X–Z).

### TPLSM Analysis

We imaged parietal cortex in KA treated mice with a custom Prairie Technologies (Madison, WI) Ultima IV *in vivo* two-photon microscope powered by a Spectra-physics DeepSee Mai Tai HP (Mountain View, CA) Titanium: Sapphire laser. The microscope was developed with an integrated intrinsic signal optical recording setup (Optical Imaging Inc.), using epi-illumination powered by a Till Photonics (GmbH, Germany) Polychrome 5000 monochromator. Image analysis followed the analysis described for fiber-optic confocal recordings and was carried out with ImageJ^38^.

### Immunohistochemical procedures

After each recording session, we overdosed the mice with Nembutal (100 mg/Kg) and fixed their brains in 4% paraformaldehyde. We cut 50 μm cryosections and every sixth hippocampus-containing section was stained for rabbit anti-AIF (1:200) (Millipore, USA), rabbit anti-active caspase-3 (1:250) (BD Pharmigen, USA), mouse anti-Neu-N (1:200) (Millipore Corporation, USA), rat anti-endoglin (CD105) (1:250) (Developmental Studies Hybridoma Bank, USA), rat anti-NG2 Chondroitin Sulfate Proteoglycan Antibodies (1:200) (Millipore Corporation, USA), or mouse anti-α-SMA (1:200) (Thermo scientific, USA) as primary antibodies. We then added sheep anti-rabbit IgG antibodies conjugated to Cy3 as secondary (1:500) (Sigma-Aldrich, USA) to stain AIF+ and Caspase-3+ cells in red; goat anti-mouse IgG conjugated to Dylight-488 (Jackson ImmunoResearch, USA) (1:500) to stain Neu-N+ cells in green; goat anti-mouse IgG conjugated to Dylight-405 (Jackson ImmunoResearch, USA) (1:500) to stain mural cells in blue; biotin-tagged goat anti-rat IgG (1:200) (ABC Elite, Vector Laboratories, Burlingame, CA) completed by avidin-biotin/CY3 tyramide signal amplification (1 h) (CY3-TSA, Perkin Elmer Life Sciences, Inc., Boston, MA, Cat#: SAT704A001EA, 1:50) to stain the vessels in the animals that we did not injected the dye; or goat anti-mouse IgG conjugated to DyLight-549 (1:500) (Jackson ImmunoResearch, USA) to stain SMA+ cells in red. Finally, we stained the sections with fluorescent DNA-binding dye, 4′,6-diamidino-2-phenylindole dihydrochloride (DAPI, Sigma-Aldrich, USA). The general protocol used for the immunohistochemistry was as follows: we first treated the tissue sections for antigen retrieval 15 minutes in phosphate-buffered saline (PBS) containing TritonX-100 0.5% (Sigma-Aldrich, USA) or, if necessary, in proteinase K (1:2 dilution, Dako, USA) in PBS 1X for 15 min, and then washed them in PBS 3 × 5 min. We blocked the sections in 80% PBS containing triton X-100 0.5%, 10% fetal bovine serum (PAA laboratories Inc., Canada), and 10% Gelatin of 2% (Sigma-Aldrich, USA) for 1 h at room temperature. We then incubated the tissue in the primary antibody at appropriate dilution in blocking solution at 4 °C overnight. We washed the sections in PBS and incubated them for 2 h at room temperature (light shielded) in secondary antibody diluted with blocking solution, and rewashed them with PBS, incubated in DAPI for 2 min, with a final wash in PBS for 10 min. Finally, we cover-slipped the sections using Prolongold Antifade reagent (Invitrogen, USA).

### Stereology methods

We counted AIF+/− cells^26^,^28^ from the 10 animals with the most consistent immunofluorescent staining (5 KO vs 5 WT; 5 KA vs 6 sham). We then analyzed a random sub-sampled group of DAPI+ cells to determine their proximity to blood vessels. This sub-sampling (maximum of three per disector) controlled for clustering that occurred in some disector locations containing as many as 17 DAPI+ neurons. We developed an algorithm in which we masked the entire stack of green fluorescent vessels and then sequentially unmasked concentric spheres in increasing steps of 2 μm radius, starting with a 2 μm radius sphere at the three-dimensional center of each sub-sampled DAPI+ nucleus (Supplementary Fig. 4). The smallest sphere that contained a green fluorescent blood vessel was determined to be the distance of the cell from the nearest vessel (maximum observed: 36 μm).

We binned each neuron-vessel distance into one of four distributions dependent on whether the animal was KO or WT (or Kainate vs. sham), and whether the neuron was AIF+ or AIF- (Figs 5 and 6A,B). We statistically tested mean differences (two-tailed Chi-square test for trend) on the raw data bins (Graphpad Prism 6.0, USA). Gaussian curve fits and normalization (to the highest raw data point in each distribution) of the data were computed solely for graphical visualization purposes.

For the trans-nuclear versus cytosolic AIF analysis (5 KO vs 4 WT animals), as well as the caspase and Neu-N analyses (3 KO vs 3 WT animals), we created images of the immunohistochemically stained sections with a Nikon Eclipse 80 microscope (Nikon Instruments, Melville, NY), equipped with a Nikon 100 W mercury light source for fluorescence illumination. A MAC5000 XYZ stage controller (Ludl Electronic Products, Hawthorne, NY) and a linear encoder (Heidenhain, Schaumburg, IL) affixed to the stage (Z movement was monitored to an accuracy of +/−0.1 μm) controlled stage movement. An average of 12 images at 2 μm Z spacing were taken from the top to bottom section surfaces for each channel separately, at an XY spacing of 450 μm systematic-randomly spaced throughout the entire CA1-3 regions. StereoInvestigator software (Microbrightfield, Williston, VT) facilitated both stage movement and stack capture. A MBF Bioscience CX9000/Microfire camera integrated with the PC/software via Firewire (Optronics, Goleta, CA) acquired high-resolution 8-bit grayscale images (1600 × 1200 pixels; 5.4 pixels/μm final magnification) in each fluorescence channel, using a Nikon PlanApo 40x/0.95NA air objective. We acquired AIF images at a constant 500 ms exposure time, whereas we adjusted exposure for DAPI (20–100 ms) and blood vessels (250–500 ms) to optimize the signal-to-noise ratio. We kept all other instrument and configuration settings constant for the duration of image acquisition. We alternated between sections from WT and KO mice to counterbalance any potential changes in the imaging conditions over time (e.g., lamp intensity). This process produced an average of 60 stacks (+/−3 s.e.m.) per subject, which we then subdivided into four (550 × 550 pixel) quadrants for further analysis. To quantify pyramidal neuron number, we counted only large round nuclei within the pyramidal cell layers of CA1–313, using the disector technique^26^ to count neurons which came into focus in a disector frame (5600 μm^2^), and then counted only those neurons within the middle 6 μm of the section (average post-processing section thickness: 22.8 μm +/−1.0). We found an average of 470 nuclei (+/−33 s.e.m.) per subject. The total DAPI+ neuron number was calculated using the fractionator technique^27^, with sampling fractions of 1/6 for the sections, 1/36 for the area, and 6/(mean thickness) in Z.

Caspase-3 expressing cells (from 3 WT and 3 KO animals), as well as cytoplasmic AIF and nuclear translocated AIF expressing cells (from 4 WT and 5 KO animals) were counted using the optical disector counting method^39^ of an image analysis system (Stereologer, Version 2.1, Stereology Resource Center, Inc, USA). This image analysis system consists of a color camera (Imi Tech, model IMC-147FT, USA), a personal computer (Dell, USA), a computer controlled motorized specimen stage for X, Y and Z-axis (Applied Scientific Instrumentation, model MS-2000, USA) and a microscope (Olympus, model BX53, USA) with a fluorescent light source (Polychrome V, TILL Photonics, Germany). For each section, we outlined the region of interest by tracing a contour around the hippocampus CA1–3 region using low magnification objective (4x). The software generated a random grid of 3-dimensional counting frames (dissector). We counted cells in each group using a 60X objective according to the unbiased counting rules of the optical dissector^39^. Previous studies determined the counting frame area, disector height, and sampling grid area. To measure the distance between caspase+ cells and vessels we marked the cells in the center of each disector using the StereoInvestigator software (MicroBrightField, Inc., USA), and created 3-dimensional reconstructions of the vessels using NeuroLucida software (MicroBrightField, Inc., USA); the distance between each cell and the nearest vessel was calculated using NeuroLucida Explorer software from the StereoInvestigator and NeuroLucida data files.

Finally, we carried out the volumetric analysis of the mural cells with Imaris software (Bitplane AG, Switzerland).

## Additional Information

**How to cite this article:** Leal-Campanario, R. *et al*. Abnormal Capillary Vasodynamics Contribute to Ictal Neurodegeneration in Epilepsy. *Sci. Rep.* **7**, 43276; doi: 10.1038/srep43276 (2017).

**Publisher's note:** Springer Nature remains neutral with regard to jurisdictional claims in published maps and institutional affiliations.

## Supplementary Material

Supplementary Information

Supplementary Movie 1

Supplementary Movie 2

Supplementary Movie 3

Supplementary Movie 4

Supplementary Movie 5

Supplementary Movie 6
