# Supplementary material for: Abnormal Capillary Vasodynamics Contribute to Ictal Neurodegeneration in Epilepsy
Source: Sci Rep. 2017 Feb 27;7:43276. doi: 10.1038/srep43276 (PMC5327474; doi:10.1038/srep43276)
Supplement: Supplementary Information [file srep43276-s1.pdf]

Abnormal Capillary Vasodynamics Contribute to Ictal Neurodegeneration in Epilepsy

Rocio Leal-Campanario[1,3,^], Luis Alarcon-Martinez[1,4,^], Hector Rieiro[1,5,^], Susana Martinez-Conde[1,2], Tugba Demirci[1], Xiuli Zhao[1,6], Jonathan LaMee[1,7], Pamela J. Osborn Popp[1,8], Michael E. Calhoun[9], Juan Ignacio Arribas[1,10], Alexander A. Schlegel[1], Leandro Luigi Di Stasi[1], Jong M. Rho[1,11], Landon Inge[12], Jorge Otero-Millan[1,5], David M. Treiman[1], and Stephen L. Macknik[1,2,*]

Supplementary Information

Detailed Methods

Anesthetized model methods

Pilot experiments to develop the surgeries and blood flow recording methods used anesthetized Kcna-null mutant animals. We were surprised to find that the mice continued to have full tonic-clonic seizures despite being under deep Ketamine-Xylazine anesthesia. This allowed us to do an initial study of hippocampal capillary blood flow dynamics in anesthetized animals (**Fig. 1B,E; Supplementary Fig. 3B,E,H,K**) before carrying out a more complete study in awake animals (**Fig. 1A,D; Supplementary Fig. 3A,D,G,J**).

We recorded from 27 anesthetized mice (12 KO and 15 WT), with an age range of postnatal day 18-60. While recording, the mouse's head was immobilized with the surgical stereotaxic apparatus. The level of anesthesia was carefully monitored and maintenance

doses of Ketamine-Xylazine were given as needed (one-third to one-half of the original dose) throughout the duration of the recording session (up to eight hours).

Kainate model animals

The non-NMDA receptor agonist kainic acid (Sigma-Aldrich; K0250) or saline control solution was injected subcutaneously with 20–30 mg/kg of kainic acid or saline control solution. Animals were placed in the recording chamber with their head fixed to allow awake intrahippocampal recording of the capillary beds and epidural EEG before kainic acid injection.

To test the kainic acid injection dose, we measured the seizure intensity after different injections for a group of animals. After the injection, we placed the animals in a clear plastic cage and we monitored their locomotor activity and EEG.

Confocal Laser Endomicroscopic imaging methods

We either recorded vasospasms with a single-band Cellvizio (Leica, USA, model FCM-1000) laser scanning microscope targeting a 488 nm laser down the bundle (beveled at the tip to penetrate the tissue) at ≥12 Hz (data in **Fig. 1, Supplementary Figs. 1-3 and 8**), or a dual-band Cellvizio (Mauna Kea Technologies, Inc, Paris France) targeting both a 488nm and a 660nm laser at ≥12 Hz (**Fig. 4A-R**). Each fiber at the beveled surface captured the emitted fluorescence and the photons were descanned into avalanche photodiode detectors

to record each movie (**see Supplementary Movie 1; Supplementary Figs. 2 and 8**). Each fiber-bundle objective contained 5000-6000 fibers.

As described elsewhere [1], seizure episodes were rated on the Racine scale and commenced with automatisms including staring, rigidity, and immobility, followed by jaw movements, blinking, head bobbing, and forelimb clonus. The next stage of seizures involved rearing, forelimb/head clonus, tonic/clonic seizures, postural imbalance, uncontrolled running, and jumping that defined the latency to first maximal seizure. In addition, we used the Racine scale [2] to score seizure activity: Stage 1, immobility; Stage 2, forelimb and/or tail extension, rigid posture; Stage 3, repetitive movements, head bobbing; Stage 4, rearing and falling; Stage 5, continuous rearing and falling; Stage 6, severe tonic-clonic seizures.

To analyze each trial's time course (**Supplementary Figs. 2 and 3,** we used custom software in MATLAB to determine four time points in each vasospasm:

1) The time at which the vasospasm constriction started, i.e. the "onset" when the signal first began to dip below baseline.
2) The time at which the constriction leveled out at its minimum, which typically occurred shortly before the termination of the vasospasm (on average at t = ~80 secs after onset).
3) The time at which the vasospasm terminated (dilation started).
4) The time at which the dilation ends, i.e. when the signal resumed its plateau.

For the onset τ, the data between 1) and 2) above were normalized to the range [0,1] and fitted to a curve of the form: $x(t) = \exp(-t/ \tau)$

For the termination tau, data between 3) and 4) above were normalized and fitted to: $x(t) = 1 - \exp(-t/\tau)$

Finally, we studied the vasodynamics and carried out stereological analyses in these animals (**Figs. 1C,F and 5K-N; Supplementary Fig. 3C,F,I,L;** see **Supplementary Table 2**).

Supplementary References

1. Schauwecker, P.E.. Seizure-induced neuronal death is associated with induction of c-Jun N-terminal kinase and is dependent on genetic background. *Brain research.* **884**, 116-128 (2000).
2. Racine, R.J. Modification of seizure activity by electrical stimulation. II. Motor seizure. *Electroencephalogr. Clin. Neurophysiol.* **32**, 281-294 (1972).
3. West, M.J., Slomianka, L. & Gundersen, H.J. Unbiased stereological estimation of the total number of neurons in thesubdivisions of the rat hippocampus using the optical fractionator. *Anat. Rec.* **231**, 482-497 (1991).

Supplementary Figures

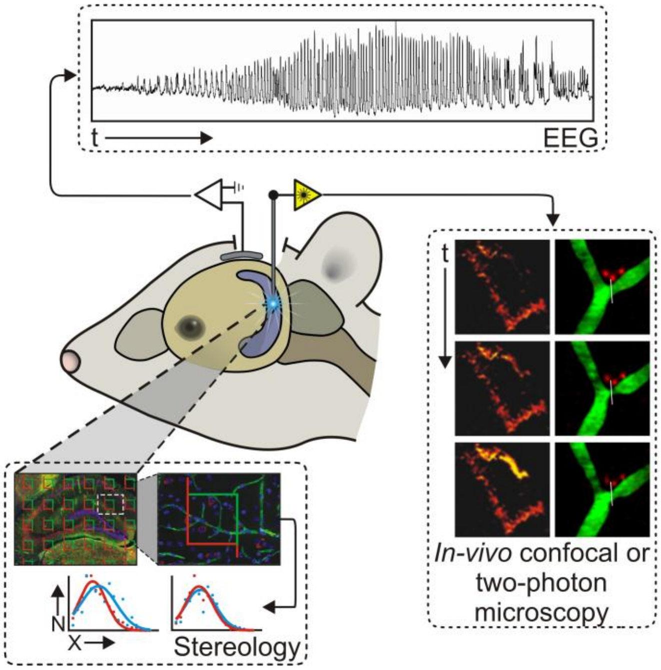

Supplementary Figure 1. Schematic of the project's methodology. We recorded EEG while conducting confocal microscopy in hippocampal capillaries of awake epileptic mice, and WT littermates, or two-photon laser scanning microscopy in cortical capillaries. Histological and stereological methods then determined that abnormal blood flow drives much of the cell death and oxidative stress caused by seizures.

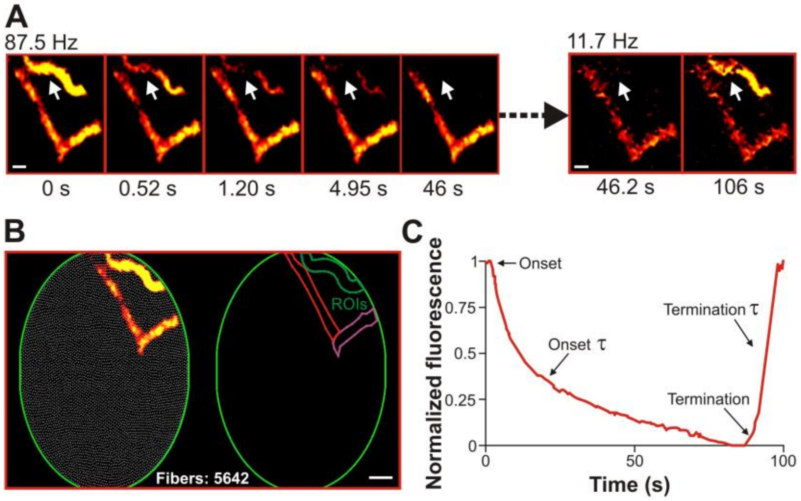

Supplementary Figure 2. Fiber-coupled laser-scanning confocal endomicroscopy of hippocampal capillaries (A) An occluded capillary in a KO animal releases from vasospasm rapidly and then slowly reconstricts over a 10 secs period. Recorded at 11.7 Hz. See **Supplementary Movie 1**. **(B)** Blood flow image with white dots overlaid to represent the position of each fiber on the bevel-cut surface of the fiber bundle (left image) and ROIs created to determine fluorescence changes over time for each vessel (right image). **(c)** Capillary fluorescence (ΔF/F) as a function of time during an ictal vasospasm, where F is fluorescence magnitude at time 0 before each vasospasm. Notice that the onset rate proceeds more slowly than the termination rate. This is presumably due to local blood pressure working against the mural cell constriction (see also **Supplementary Figure 8**). The vessel opens more rapidly at the termination of the vasospasm. Scales for panel a= 10 µm, for panel b= 20µm. See **Supplementary Movie 1**.

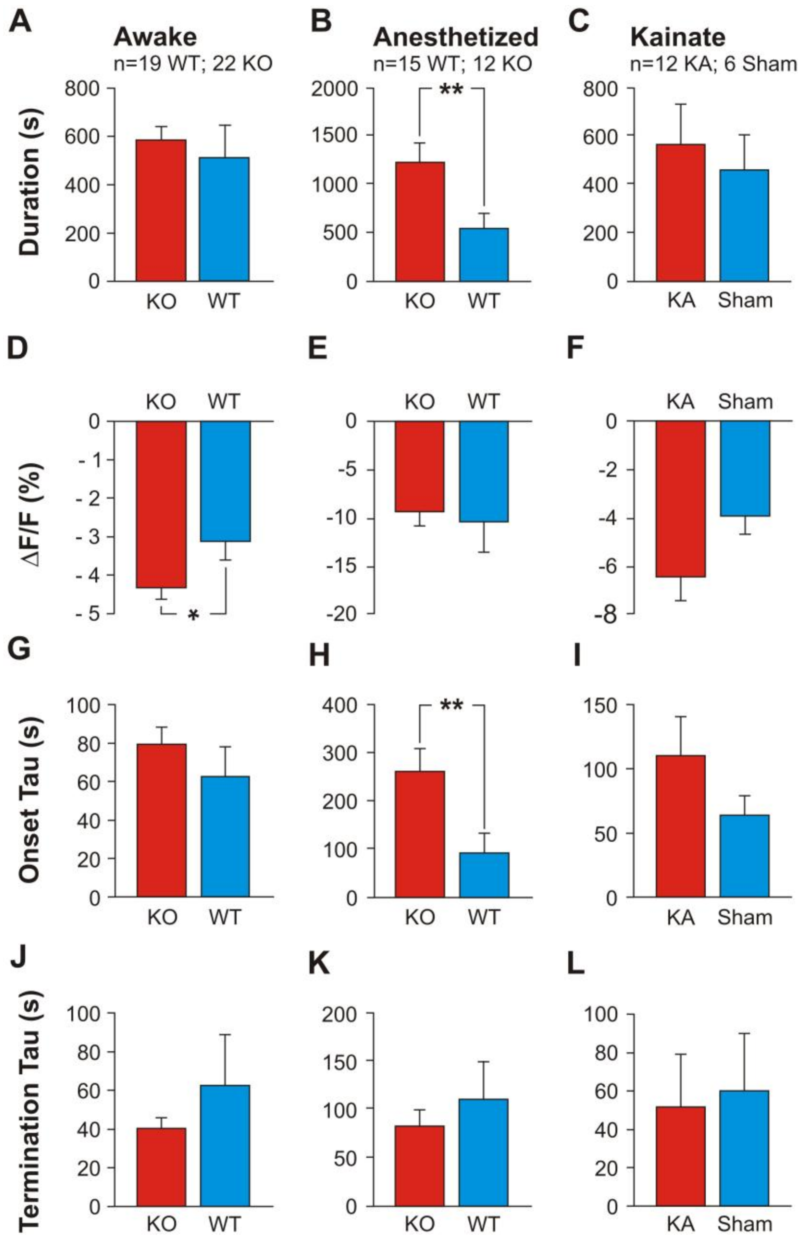

Supplementary Figure 3. KO vs WT vasodynamics in awake (A, D, G and J) and anesthetized Kv1.1 KO versus WT littermates (B, E, H and K) and Kainate vs sham (C, F, I and L) mice. (A-C) Ave. vasospasm duration. **(D-F)** Ave. vasospasm magnitude measured as a change in magnitude of fluorescence as a function of time. **(G-I)** Ave. vasospasm onset speed. **(J-L)** Ave. vasospasm termination speed. For all panels: *= significant difference ($p<0.05$), ** ($p<0.01$), **** ($p<0.0001$). Related to **Figure 1.**

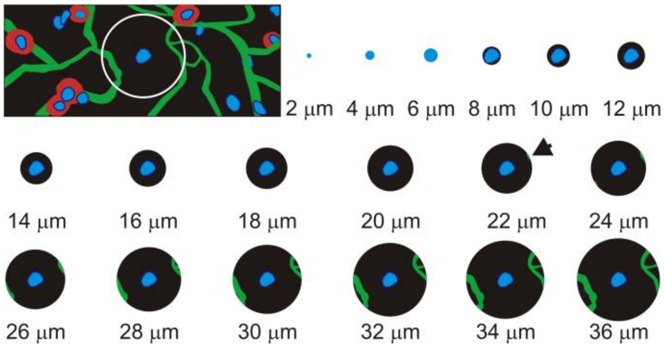

Supplementary Figure 4. The logic of our novel 3D stereological method to determine cell-vessel distance. The blue spot in the cartoon depicts a DAPI+ pyramidal cell nucleus, as viewed from the top of a 3D reconstructed image stack. The white circle in the cartoon represents the radius of the largest sphere (36 μm) used in the analysis. The uppermost left 2 μm sphere is centered three-dimensionally at the center of the selected DAPI+ nucleus. The sphere becomes sequentially larger in 2 μm radius steps, and eventually unmasks the green fluorescein-stained vessel within the stack (at 22 μm in this example, see arrow). The distance of the nearest blood vessel to the central DAPI+ nucleus was determined as the radius of the smallest sphere that contained a vessel. We binned the cell counts by distance as a function of both cohort (KO vs WT), and whether they were AIF+/-. These cell counts created the distance measurements in **Figures 3I, 5G-N and 6C-D**.

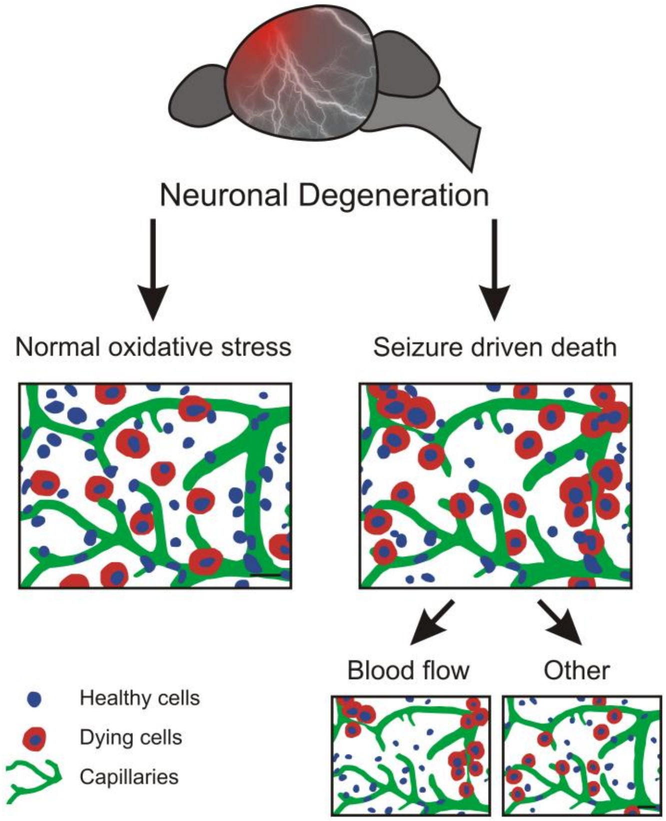

Supplementary Figure 5. Proposed model of neuronal degeneration in epilepsy. Neural degeneration can occur due to normal oxidative stress (non-epileptic sources of cell death), or through epilepsy. Epilepsy can further cause a macroscopically hyperemic seizure focus that leads to both cell death from hypoxic apoptosis associated with abnormal vasospasms near ischemic vessels, or from other types of cell death (i.e. excitoxicity) in which cell distances from vessels are spatially random. The combined result is that AIF+ cells tend to be nearer to blood vessels than (randomly distributed) AIF- cells. Scale bars 10 μM.

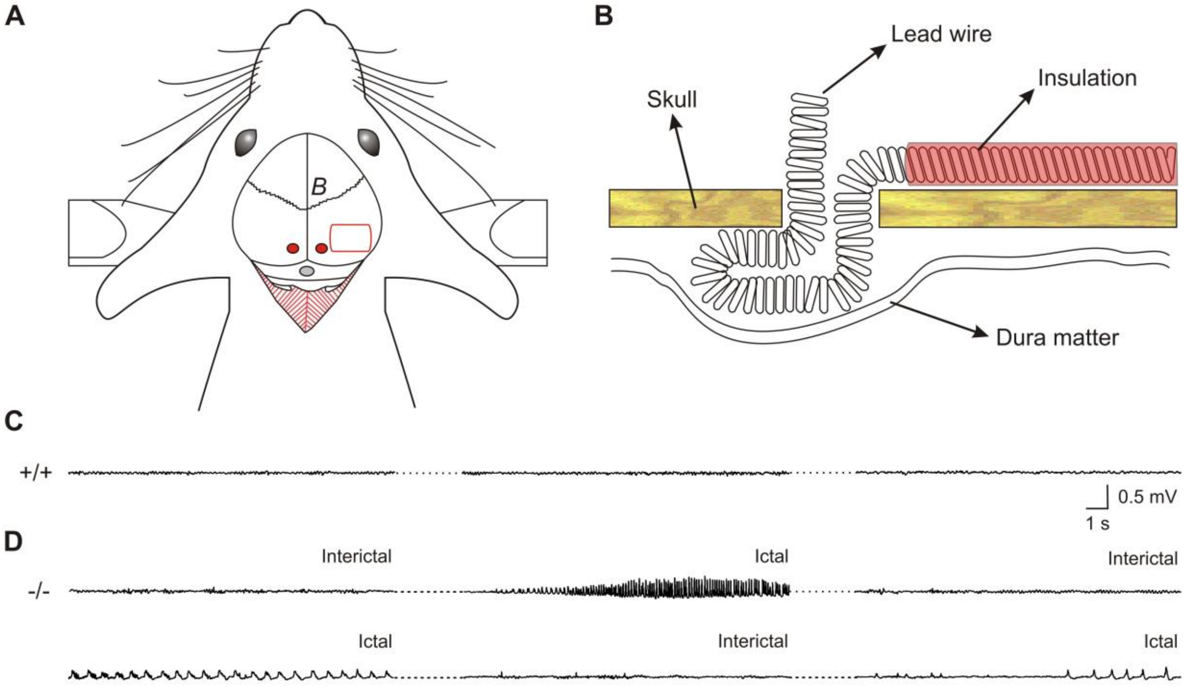

Supplementary Figure 6. Surgical preparation for the implantation of the electroencephalographic (EEG) activity recording system (A,B) and typical spontaneous EEG activity recorded with the implantable radiotelemetry transmitter in awake KO and WT mice (C,D), related to Figure S2. (A) Diagram of the mouse head positioned on the stereotaxic device, showing skull perforations performed to insert EEG recording electrodes (red and grey dots) as well as the craniotomy for the fiber-optic bundle used in the confocal microscopy (red rectangle). **(B)** Illustration of the coiled lead wire in contact with the dura for detection of EEG. *B*, *Bregma* point. We recorded a total of 67 seizures in our awake KO animals (none in WTs), with an average of 3.35 (+/- 0.79) seizures per animal, occurring at a rate of 0.58 (+/- 0.13) seizures per hour. (C,D). **(C)** Spontaneous EEG activity in WT mice. **(D)** Spontaneous ictal and interictal epileptiform activity in KO mice.

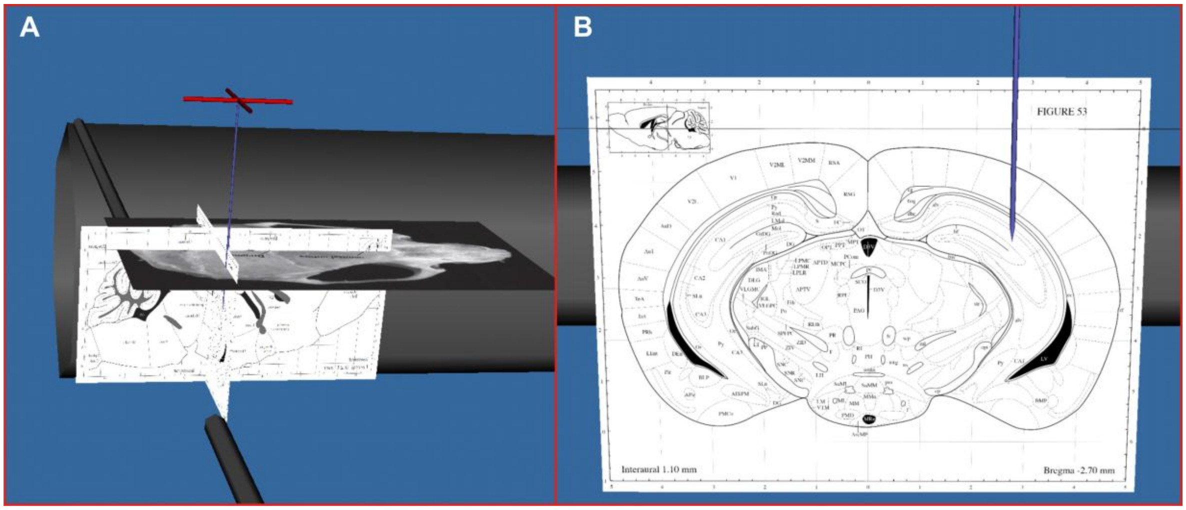

Supplementary Figure 7. StereoDrive 3D software atlas view. Related to Supplementary Figures 1, 2 and Supplementary Movies 1 and 2. (A) Sagittal view of the atlas and probe positioned for recordings. **(B)** Coronal view. Stereotactic coordinates of recording: Anterior-Posterior= 2.70 mm posterior to *Bregma*; Lateral= 2.70 mm to the right side from the midline.

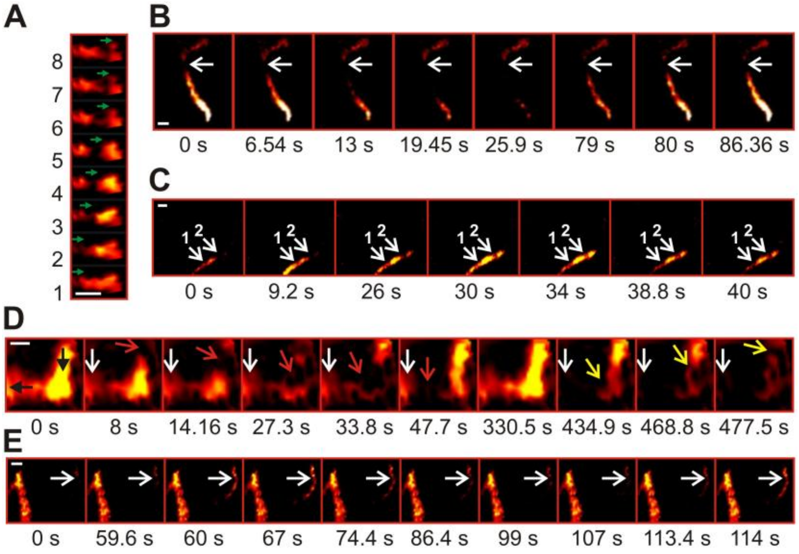

Supplementary Figure 8. Fiber-coupled laser-scanning confocal imaging of hippocampal capillaries. (A) Eight sequential images of a length of capillary, recorded at 87.5 Hz (42 in main text). Arrows indicate a pair of red blood cells (black) flowing as a cluster to the right at 219 μm/s. **(B)** A capillary vasospasm in a KO progresses in a typical fashion. Recorded at 11.7 Hz. **(C)** An occlusion of a capillary due to a mural cell contraction in a KO mouse. Arrows point to mural cells (1 and 2). Mural cell 2 at first causes the vasospasm, and then releases at ~26 secs. **(D)** High-speed (87.5 Hz) analysis of ictal vasospasms leading to red blood cell blockages in a capillary network. First frame (left) shows serum flow through the vessel network (black arrows). Eight seconds later, a vessel constriction appears (white arrow), due to an external (unstained) mural cell. Red blood cells clog the anastomosis (red arrows) over the following 40 s. The vessel then reopens and flows freely for ~5 minutes, followed by a second constriction event and a red blood cell blockage throughout the local network (yellow arrows). **(E)** Vasospasm of a WT mouse capillary (white arrow points to a mural cell shadow in the capillary), which begins the recording in vasospasm, releasing at time 60 secs, then vasospasming again at time ~86 secs, to release again at time ~114 secs. Scales for panel A-C= 10 μm; Scale for panel D= 5 μm; Scale for panel E= 20 μm. See also **Supplementary Figure 2**.

Supplementary Tables

	Subject Cohort	Total Number	CE* (number)	DAPI+ Cells	N of Sections	Thickness (μm)
1	KO	471303	0.043	748	9	17.41
2	KO	423425	0.082	471	10	24.84
3	KO	362218	0.061	416	8	24.06
4	KO	473834	0.082	457	9	28.65
5	KO	308598	0.069	351	8	24.29
6	WT	330920	0.119	452	8	20.23
7	WT	321445	0.196	409	9	21.72
8	WT	370354	0.063	459	7	22.29
9	WT	367328	0.058	482	9	21.06
10	WT	394038	0.066	456	8	23.88
	Mean:	***382346***	***0.084***	***470***	***8.500***	***22.84***

*Coefficient of Error [3].

Supplementary Table 1. Hippocampal regions CA1-3 for stereological analyses. We analyzed two cohorts (KO and WT mice; second column from the left) and we measured the total number of cells counted stereologically in each category (third column from the left). The coefficient of error (CE; third column) represents the precision of a population size estimate. The total number of DAPI+ cells (fourth column) was calculated using the fractionator technique [3]. The section thickness (final column) is the average thickness after histological processing.

	Subject Cohort	Total No AIF+ Cells	CE*	AIF+ Cells	Total No AIF- Cells	CE*	AIF- Cells	Total No Cells	CE*	N of Sections	Thickness (µm)
1	Sham	36800	0.053	138	361067	0.020	1354	397867	0.017	9	32.10
2	Sham	37164	0.064	146	248436	0.024	976	285600	0.021	11	30.10
3	Sham	47163	0.051	147	481250	0.020	1500	528413	0.017	8	36.00
4	Sham	202650	0.058	772	296888	0.022	1131	499538	0.019	8	33.00
5	Sham	44800	0.065	240	203467	0.025	1090	248267	0.021	10	33.20
6	Sham	194571	0.069	681	246286	0.026	862	440857	0.023	7	36.00
	Mean:	***93858***	***0.060***	***354***	***306232***	***0.023***	***1152***	***400090***	***0.020***	***8.800***	***33.40***
7	KA	110473	0.018	454	256473	0.009	1054	366947	0.010	10	37.20
8	KA	81389	0.021	293	232778	0.010	838	314167	0.012	9	34.40
9	KA	71467	0.026	268	172533	0.013	647	244000	0.015	7	32.00
10	KA	94500	0.021	405	214667	0.011	920	309167	0.012	9	32.60
11	KA	50167	0.022	215	217700	0.011	933	267867	0.012	9	32.80
	Mean:	***81599***	***0.021***	***327***	***218830***	***0.011***	***878***	***300429***	***0.012***	***8.800***	***33.80***

*Coefficient of Error [3]

Supplementary Table 2. Hippocampal regions CA1-3. Comparison between sham and kainate animals. We analyzed two cohorts (sham and KA mice; first column) and we measured the total number of AIF+ cells (second column) as well as the total number of AIF- cells (fifth column) using standard stereological cell counting methods. The coefficient of error (CE; third, sixth and ninth columns) represents the precision of a population size estimate. The total number of AIF+ cells (fourth column) and AIF- cells (seventh column) were calculated using the fractionator technique [3]. The section thickness (eleventh column) was measured after histological processing. *Coefficient of Error [3].

Supplementary Movies

Click on images to see the video

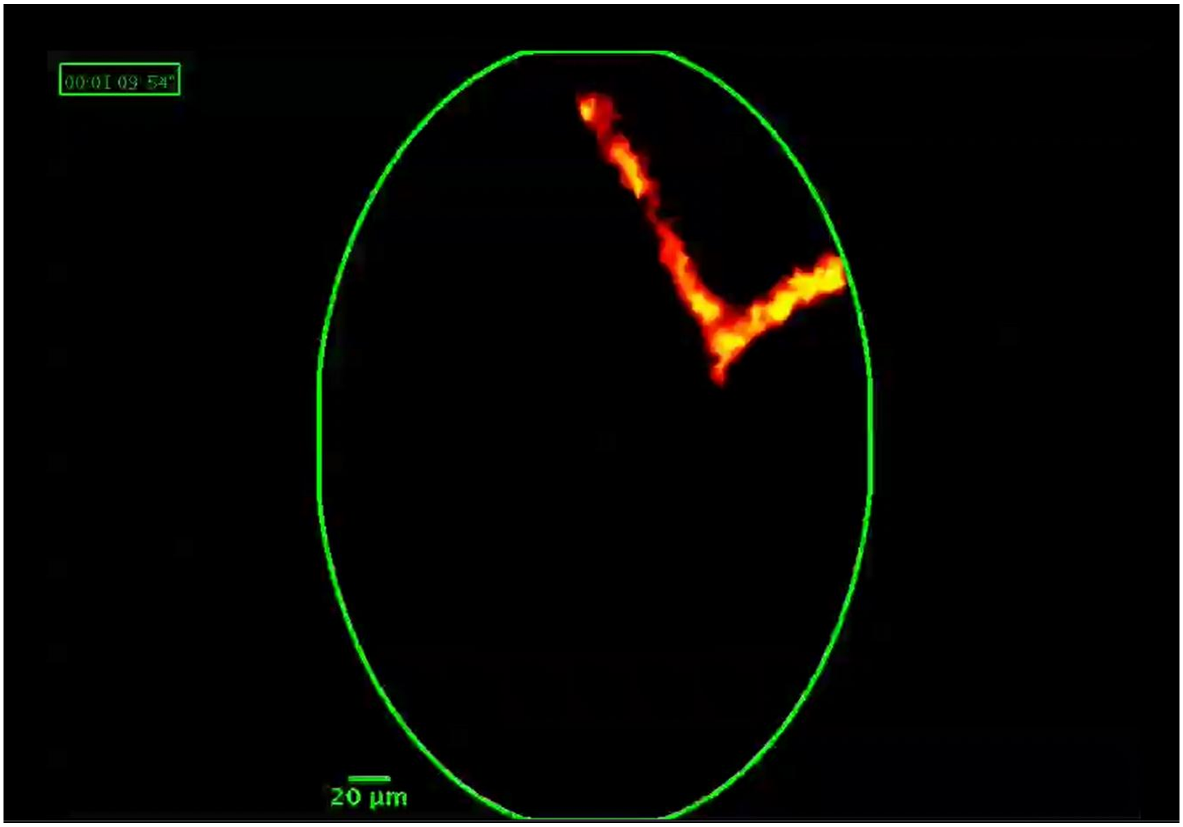

Supplementary Movie 1. Blood flow and vasospasm imaging of vessel in Supplementary Figure 2A. An occluded capillary in an epileptic Kv1.1 mutant mouse releases from vasospasm rapidly and then slowly reconstricts over a 10 s period, recorded at 11.7 Hz.

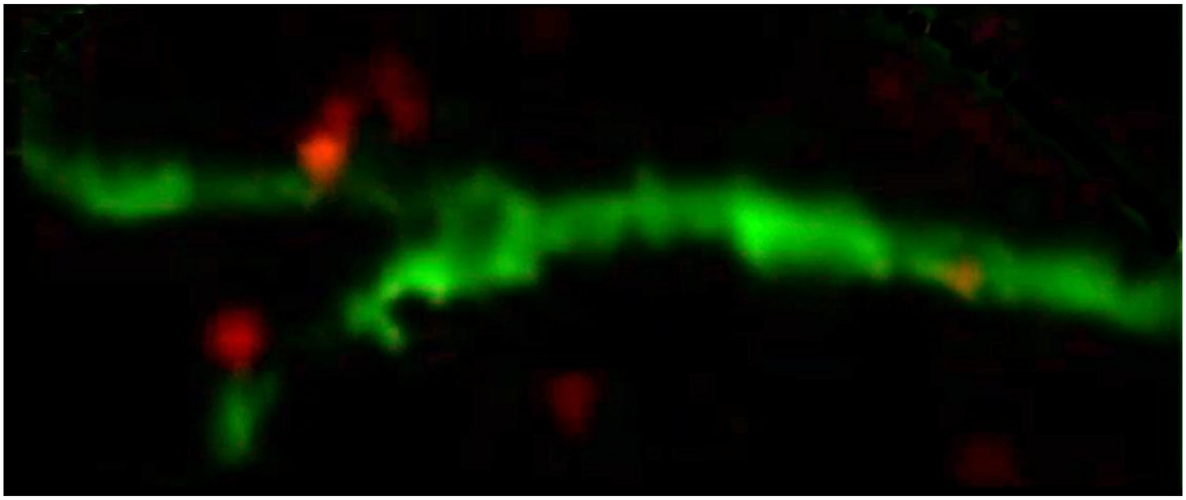

Supplementary Movie 2. Blood flow and vasospasm imaging of vessel in Figure 4A-H.

Vasospasm and mural cell recorded (11.7 Hz) in a KO hippocampal capillary.

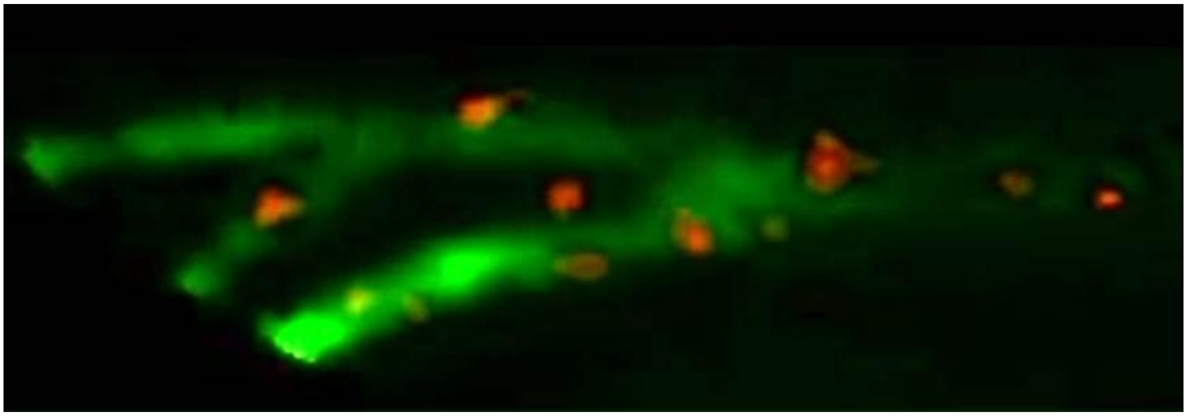

Supplementary Movie 3. Blood flow and vasospasm imaging of vessel in Figure 4I-R.

Vasospasm, leukocyte blockages, and mural cells recorded (11.7 Hz) in a WT hippocampal capillary.

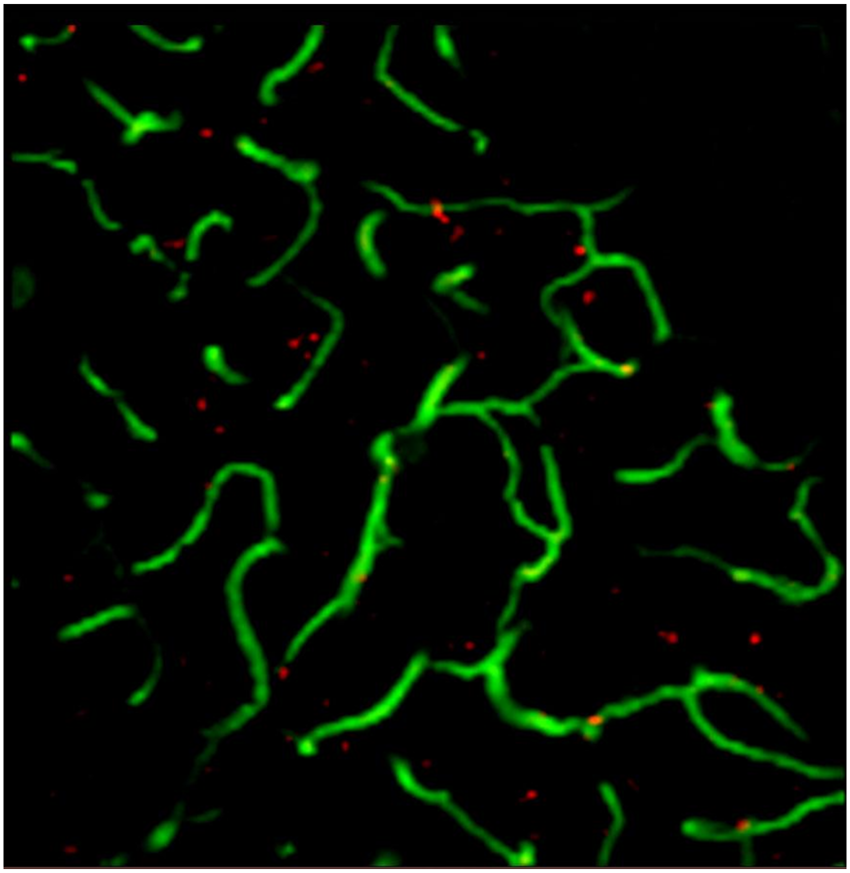

Supplementary Movie 4. In vivo TPLSM 60 µm deep volumes recorded of non-uniform blood flow in WT mouse parietal cortex from Figure 4S-W.

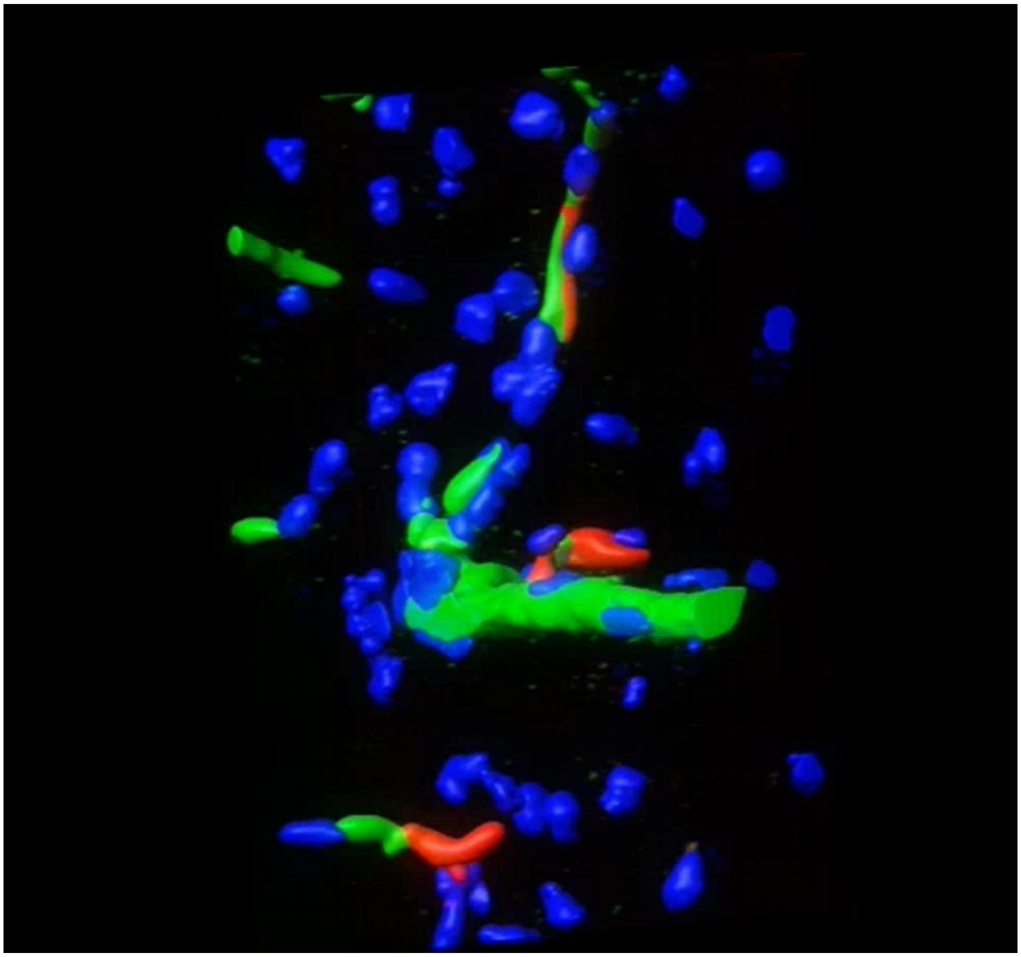

Supplementary Movie 5. Digital 3D modeling of the action of mural cells on capillaries. The stack of optical sections from **Figure 3F-H** was digitally segmented for nuclei (blue), capillaries (green), and α-SMA antibody labeled mural cells (red). 3D models were created from these data, and the central mural cell from **Figure 3F-H** was enhanced and magnified. Digital analysis reveals that the mural cell completely surrounds the capillary at the point of constriction, and that the vessel is constricted to an approximate diameter of 1 µm. The mural cell extends down the occluded vessel but the green serum fluorescence is completely cutoff mid-mural cell, indicating that this mural cell completely blocked serum flow during this vasospasm.

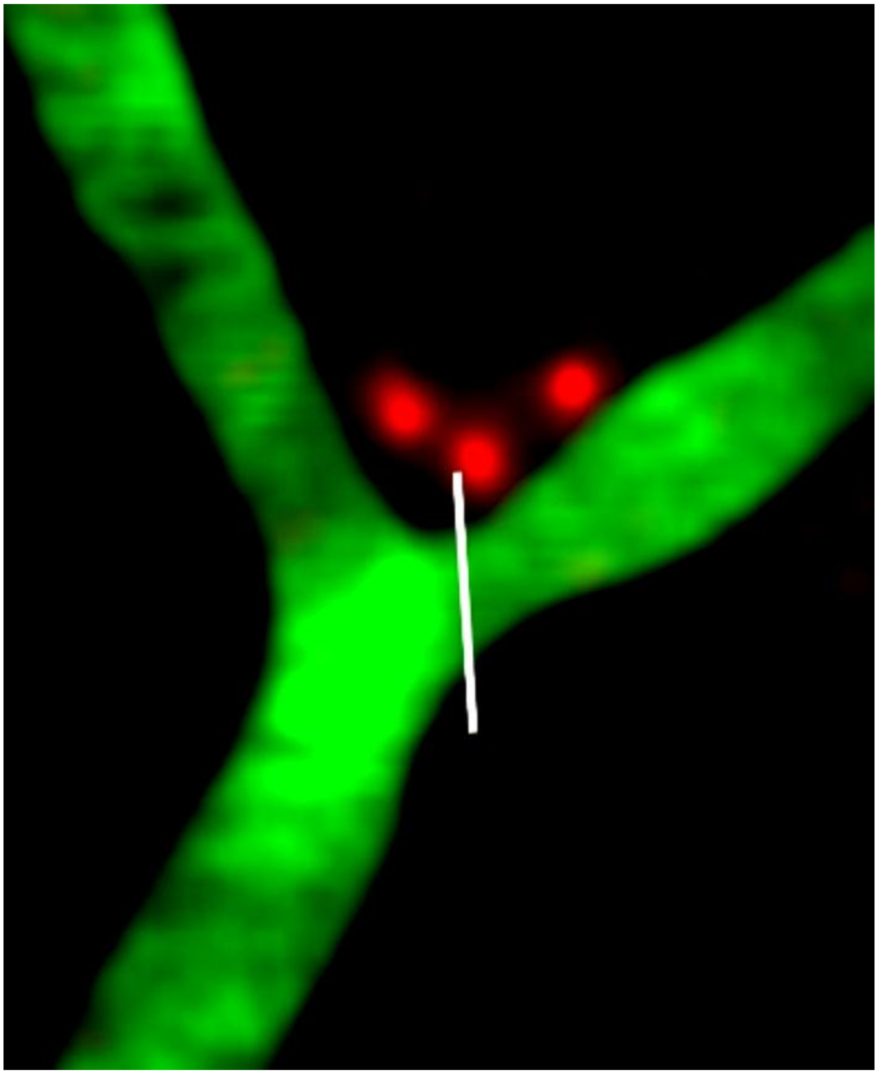

Supplementary Movie 6. In vivo TPLSM of mural cell-driven (red) capillary (green) constriction from parietal cortex of KA treated seizing animal from figure 4X-Z.
